# Comparison of Research Spending on New Drug Approvals by the National Institutes of Health vs the Pharmaceutical Industry, 2010-2019

**DOI:** 10.1001/jamahealthforum.2023.0511

**Published:** 2023-04-28

**Authors:** Ekaterina Galkina Cleary, Matthew J. Jackson, Edward W. Zhou, Fred D. Ledley

**Affiliations:** 1 Center for Integration of Science and Industry, Bentley University, Waltham, Massachusetts; 2 Exponent, Inc; 3 Department of Mathematical Sciences, Bentley University, Waltham, Massachusetts; 4 Department of Natural and Applied Sciences, Bentley University, Waltham, Massachusetts; 5 Department of Management, Bentley University, Waltham, Massachusetts

## Abstract

**Importance:**

Government and the pharmaceutical industry make substantive contributions to pharmaceutical innovation. This study compared the investments by the National Institutes of Health (NIH) and industry and estimated the cost basis for assessing the balance of social and private returns.

**Objectives:**

To compare NIH and industry investments in recent drug approvals.

**Design, Setting, and Participants:**

This cross-sectional study of NIH funding associated with drugs approved by the FDA from 2010 to 2019 was conducted from May 2020 to July 2022 and accounted for basic and applied research, failed clinical candidates, and discount rates for government spending compared with analogous estimates of industry investment.

**Main Outcomes and Measures:**

Costs from the NIH for research associated with drug approvals.

**Results:**

Funding from the NIH was contributed to 354 of 356 drugs (99.4%) approved from 2010 to 2019 totaling $187 billion, with a mean (SD) $1344.6 ($1433.1) million per target for basic research on drug targets and $51.8 ($96.8) million per drug for applied research on products. Including costs for failed clinical candidates, mean (SD) NIH costs were $1441.5 ($1372.0) million per approval or $1730.3 ($1657.6) million per approval, estimated with a 3% discount rate. The mean (SD) NIH spending was $2956.0 ($3106.3) million per approval with a 10.5% cost of capital, which estimates the cost savings to industry from NIH spending. Spending and approval by NIH for 81 first-to-target drugs was greater than reported industry spending on 63 drugs approved from 2010 to 2019 (difference, −$1998.4 million; 95% CI, −$3302.1 million to −$694.6 million; *P* = .003). Spending from the NIH was not less than industry spending considering clinical failures, a 3% discount rate for NIH spending, and a 10.5% cost of capital for the industry (difference, −$1435.3 million; 95% CI, −$3114.6 million to $244.0 million; *P* = .09) or when industry spending included prehuman research (difference, −$1394.8 million; 95% CI, −$3774.8 million to $985.2 million; *P* = .25). Accounting for spillovers of NIH-funded basic research on drug targets to multiple products, NIH costs were $711.3 million with a 3% discount rate, which was less than the range of reported industry costs with 10.5% cost of capital.

**Conclusions and Relevance:**

The results of this cross-sectional study found that NIH investment in drugs approved from 2010 to 2019 was not less than investment by the pharmaceutical industry, with comparable accounting for basic and applied research, failed clinical trials, and cost of capital or discount rates. The relative scale of NIH and industry investment may provide a cost basis for calibrating the balance of social and private returns from investments in pharmaceutical innovation.

## Key Points

**Question:**

How does National Institutes of Health (NIH) investment in pharmaceutical innovation compare with investment by the pharmaceutical industry?

**Findings:**

In this cross-sectional study of 356 drugs approved by the US Food and Drug Administration from 2010 to 2019, the NIH spent $1.44 billion per approval on basic or applied research for products with novel targets or $599 million per approval considering applications of basic research to multiple products. Spending from the NIH was not less than industry spending, with full costs of these investments calculated with comparable accounting.

**Meaning:**

The results of this cross-sectional study suggest that the relative scale of NIH and industry investment in new drugs may provide a basis for calibrating the balance of social and private returns from these products.

## Teaser

This cross-sectional study examines National Institutes of Health and pharmaceutical industry investments in recent drug approvals.

## Introduction

Private sector investment and returns are classically viewed as the primary driving force for innovation. Evidence also shows that public sector investments in basic and applied biomedical research, including those from the National Institutes of Health (NIH), contribute substantively to the emergence of new drugs^1,2,3,4,5,6,7^ and drug-related patents.^2,4,8,9^ Recent economic studies have recognized the government’s contributions to pharmaceutical innovation by contextualizing government as an “early-stage investor and government funding for research as an investment.”^10,11,12,13,14,15,16,17^ As such, these studies argued that there should be an equitable balance of investment risk and return between the public and private sectors^15,16^ and framed policy regarding the pharmaceutical industry’s drug pricing practices and profits as shaping this balance.^17,18^

The objective of this study was to compare NIH investment in the products approved by the US Food and Drug Administration (FDA) from 2010 to 2019 with reported levels of investment by the industry.^19,20,21^ This comparison required an accounting for NIH spending comparable with that used to estimate total industry investment. This typically includes not only costs associated with approved products, but also costs associated with products that fail in clinical development and the cost of capital, or opportunity cost, associated with these investments.^19,20,22,23^

Funding from the NIH for pharmaceutical innovation has been estimated from total NIH budget allocations^24^ or categorical funding from the Research, Condition, and Disease Categories or Research Portfolio Online Reporting Tools (REPORTER).^25,26^ These methods do not delineate spending associated with individual products. Case study methods have been used to identify NIH contributions associated with specific patents^2,8^ or products.^5^ These methods may not capture funding for basic research, which represents half of NIH funding and is classically undertaken “without specific applications towards processes or products in mind.”^27^

Other studies have focused on NIH funding for published research associated with approved drugs or their targets.^6,28,29^ In these studies, the costs of NIH-funded projects (grants) supporting research on a drug or its target were used as a measure of the NIH contribution to that product. In this method, drug-related publications represent applied research, and those associated with the drug’s target, but not the drug, represent basic research. Initial studies using this method identified NIH funding for research underlying each of the 210 drugs approved from 2010 to 2016, with total NIH costs of more than $100 billion and funding for each first-in-class drug of more than $800 million.^28^ These studies also demonstrated spillover effects in which NIH spending for basic research in immunology or endocrinology contributed to the development of products for treating cancer.^29^

This study extended these methods by developing an accounting for NIH spending that was comparable with reported investments by the industry. Using a data set of drugs approved from 2010 to 2019 (before the COVID-19 pandemic), this analysis estimated the NIH investment in these drugs, including the cost of published basic and applied research associated with these products, cost of phased clinical trials of failed product candidates, and opportunity cost, using discount rates recommended for government spending.^30,31^ These estimates were used to compare NIH and industry investments in new drug approvals, the cost savings to the industry provided by NIH spending, and the economic efficiencies created through spillovers of NIH-funded basic research on drug targets to multiple products. These results are discussed in the context of policy regarding drug pricing and corporate profit that affects the balance of investment risk and reward between the public and private sectors.

## Methods

### Study Design

This cross-sectional study analyzed NIH-funding for published research related to drugs approved from 2010 to 2019 or their biological targets that was conducted from May 2020 to July 2022. This study did not involve human participants and was not subject to institutional review board review. The study was reported in accordance with the Strengthening the Reporting of Observational Studies in Epidemiology (STROBE) reporting guidelines.

### Data Sources

The core data collection of PubMed publications, NIH-funded projects and project costs associated with drugs approved from 2010 to 2019 has been previously described.^6^ Products approved by the FDA from 2010 to 2019 (new drug application or biologics license application [type 1]), excluding those derived from blood or tissue, diagnostic agents, vaccines, and antimicrobials, and dates of first approval were identified from annual FDA reports.^32,33^ Drug targets were identified from published literature^34,35^ or the Therapeutic Targets Database.^36^

Publications from 1960 to 2020 were identified in PubMed. Projects funded by the NIH from 2000 to 2020 were identified using the NIH REPORTER application programming interface. Projects were identified by NIH project number comprising the activity code, awarding institute, and number. Data on each project included the start year, end year, and costs for each fiscal year, subproject, or supplemental award. The analysis included phase-specific clinical success rates,^19^ average NIH costs for phased clinical trials,^37^ average industry investments,^19,20^ and drug-specific industry costs.^20^

### Derivation of NIH Costs

Funding from the NIH for publications (PMIDs) associated with study drugs or their targets was identified in NIH REPORTER using methods described previously^6,28^ (eMethods in Supplement 1). Briefly, PMIDs were identified in PubMed using optimized search terms for drugs (eTable 1A in Supplement 1) or targets (eTable 1B in Supplement 1) as well as automatic term mapping protocols, including medical subject heading terms and Boolean modifiers. The PMIDs were indexed by PubMed Identifier, publication year, and search terms. Projects funded by the NIH that were associated with PMIDs were identified using the REPORTER publication link tables. The PMIDs were further associated with 1 fiscal year of project funding (project year) and total project costs for the year corresponding to the publication year. Project years and costs were not assigned to PMIDs published after the product’s first FDA approval, before the project start year, or more than 4 years after the project end year. Drug-specific costs were calculated from 2000 through the date of first FDA approval. To account for lags between funding and publication,^38^ PMIDs with publication dates 1 to 4 years after the project end year were associated with the project end year. The PMIDs identified by drug search were categorized as applied research, which included development. The PMIDs identified by target search, but not a drug search, were categorized as basic research. Project years and costs were categorized as applied research if 1 or more PMIDs associated with that project year were identified by drug search and categorized as basic research if none of the associated PMIDs were identified in drug searches. Duplicate PMIDs, project years, and costs were eliminated independently for each calculation.

The first drug associated with a novel biological target was classified as first to target.^34,35,39^ Applied research costs were estimated from costs identified through the drug search. Basic research costs were estimated from costs identified in searches for targets of first-to-target drugs. Averages were calculated after 95th percentile outlier elimination to account for searches with poor specificity. The average number of drugs per target was recalculated from Santos et al^35^ after excluding products derived from blood or tissue, diagnostic agents, vaccines, and antimicrobials. Spending from the NIH on failed clinical trials was estimated from phase transition rates^19^ and phase-specific NIH costs.^37^ Compounded 3% or 7% discount rates^30,31^ or a 10.5% cost of capital^19^ were calculated from the project year to first FDA approval.

### Statistical Methods

Product-specific costs were compared for 81 first-in-class drugs with NIH costs estimated in this analysis and 63 drugs with industry costs described by Wouters^20^ using univariate regression in which* Cost_i _=_ _*β_0_*_ _+_ _*β_1_* Source_i_* in which *Cost_i_* is the estimated NIH cost for research associated with the product or reported industry costs; *Source_i_* is an indicator variable with a value of 0 for NIH costs and 1 for industry costs; β_0_ estimates the median and 95% CI for NIH spending; and β_1_ estimates the median and 95% CI for the difference between NIH and industry spending. Costs were inflation-adjusted to 2018. Analyses were performed in Excel (Microsoft), PostgreSQL (PostgreSQL Global Development Group), or Python. All tests were 2 tailed. A 2-sided *P* < .05 was considered statistically significant.

## Results

### Descriptive Data

The FDA approved 356 drugs from 2010 to 2019, including 336 associated with 217 known targets. PubMed searches for drug names identified 229 000 PMIDs, while searches for known drug targets identified 1.9 million publications, of which 21.4% had NIH funding (Table 1). Funding from the NIH funding was identified in 310 of 356 drug searches (87%) and in all 217 target searches (Table 1). Overall, this analysis identified NIH-funded research associated with 354 of 356 products (99.4%) approved from 2010 to 2019. The products without NIH funding were a chelating agent and osmotic laxative.

**Table 1.** NIH Funding for Basic and Applied Research Associated With 356 NMEs Approved by the FDA, 2010-2019

Characteristic	No. (%)		
	Drug search^a^	Target search^b^	Total
PubMed search results, No.			
Searches	356	217	NA
Publications in PubMed (1985-2019)	229 401	1 911 507	2 017 408^c^
REPORTER NIH-funded publications			
Publications with NIH funding (1985-2019)	36 195 (16)	409 123 (21.4)	424 293 (21)^c^
Totals			
Searches identifying publications with NIH funding	310 (87)	217 (100)	NA
REPORTER project years and costs	Applied research^d^	Basic research^d^	Total
No. of project years	42 549	317 354	359 903
Project years costs (millions), $	30 954	156 429	187 383
Total NIH funding, %	17	83	NA

Abbreviations: FDA, US Food and Drug Administration; NA, not applicable; NIH, National Institutes of Health; NME, new molecular entity; REPORTER, Research Portfolio Online Reporting Tools.

^a^ PubMed search performed with drug name and synonyms.

^b^ PubMed search performed with name of biological target.

^c^ Total is nonadditive due to publications identified in drug and target searches.

^d^ Publications identified in a drug search are classified as applied research. Publications identified in a target search, but not a drug search, are classified as basic research.

**Figure 1.**
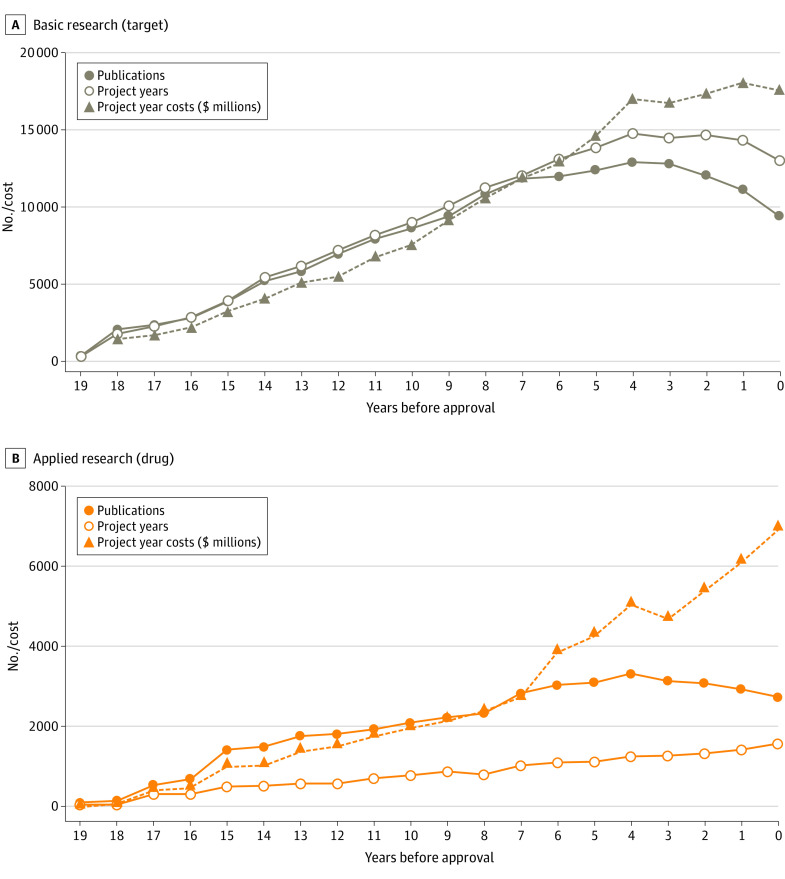
National Institutes of Health (NIH) Funding for Basic and Applied Research Associated With Drugs Approved From 2010 to 2019 by Year Before First Approval Publications (PMIDs), NIH-funded project years, and project year costs for basic research on 87 novel drug targets with first-to-target drugs (A) and for applied research on 356 drugs (B) approved from 2010 to 2019. Data are shown without applied discount rates.

Funding from the NIH totaled $187 billion; $31 billion (17%) represented applied research on approved drugs, and $156 billion (83%) represented basic research on drug targets (Table 1). Figure 1 shows annual publications, NIH project (funding) years, and costs leading to first FDA approval.

Research projects and research-related programs, which typically support investigator-initiated research, provided 40.2% of NIH funding, including 43.2% of basic research costs and 24.8% of applied research costs. However, research program projects and centers as well as cooperative agreements (including clinical translational science awards), which typically contribute infrastructure or shared research capabilities, comprised 46.2% of total costs, 42.4% of basic research costs, and 65.6% of applied research costs (eFigure 1 in Supplement 1).

### NIH Investment in Basic Research on Novel Targets

Of the 356 approvals, 86 (24.2%) were first-to-target products. Figure 1A shows NIH-funded publications, project years, and NIH costs associated with these targets leading to first-to-target product launch. Funding from the NIH was identified for all 86 targets (eTable 2 in Supplement 1).

**Figure 2.**
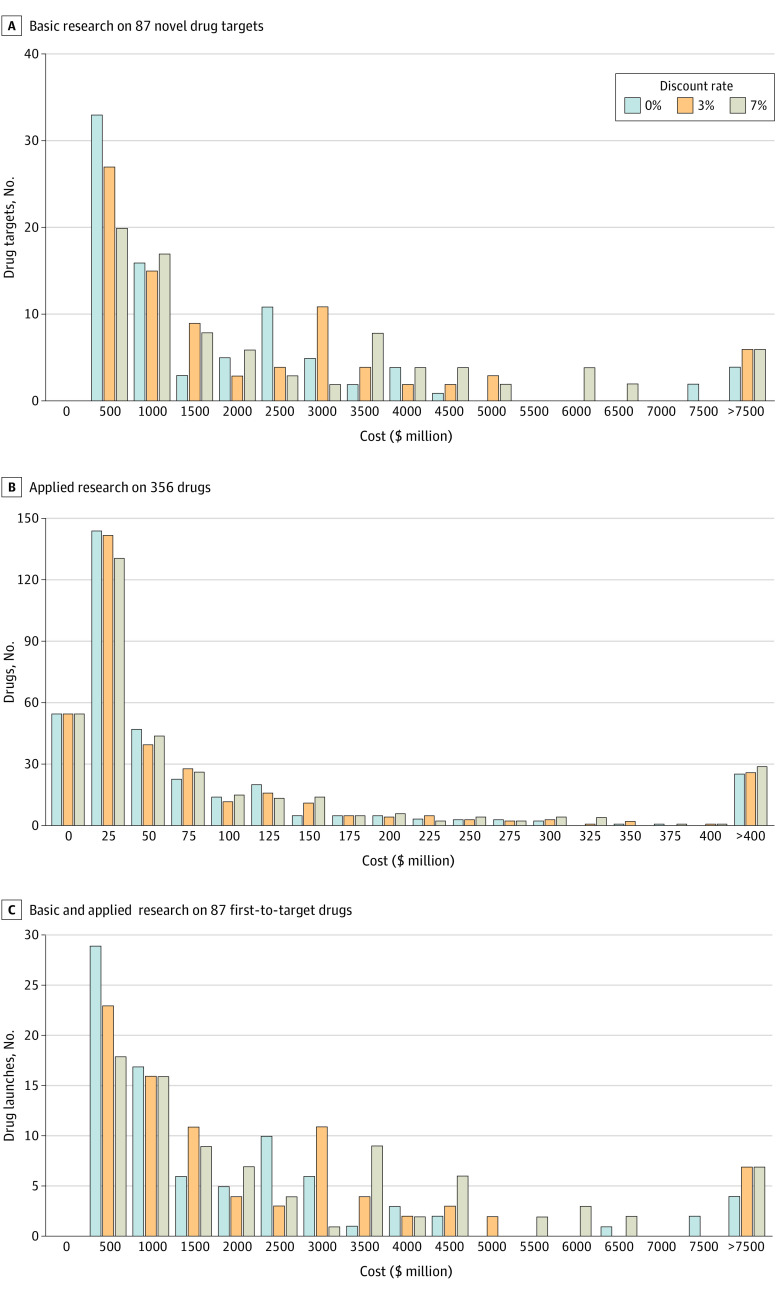
National Institutes of Health (NIH) Costs for Basic and Applied Research Associated With Drugs Approved From 2010 to 2019 All costs were calculated to the year of drug approval with no discount rate or discount rates of 3% or 7% for years before approval. A, Costs for the NIH for basic research on 87 novel targets before the launch of a first-to-target product identified by searching for a drug target, but not the drug itself. B, Costs for the NIH for published applied research on 356 drugs identified by searching for the drug. C, Costs for the NIH for basic and applied research associated with 87 first-to-target drugs.

Figure 2A shows NIH costs per novel drug target with no discount rate or 3% and 7% discount rates. After 95th percentile outlier elimination, the mean (SD) NIH cost for research on a novel drug target before a first-to-target product approval was $1.34 ($1.43) billion (3% discount, $1.63 [$1.74] billion; 7% discount, $2.15 [$1.66] billion; 10.5% cost of capital, $2.85 [$3.15] billion) (Table 2). Outliers included searches for CD-4, B-cell lymphoma 2, and epidermal growth factor receptor, which returned publications not explicitly associated with the drug target. Calculations without outlier elimination are shown in eTable 3 in Supplement 1.

**Table 2.** NIH Costs for Basic and Applied Research Associated With NMEs Approved From 2010 to 2019

Characteristic	$^a^			
	Mean discount rate (SD)^b^			Cost of capital^b^
	NA	3%	7%	10.5%
Average NIH cost to launch of first drug associated with novel drug targets (n = 86)^c^				
Basic, applied research, phased development failures^d^	1441.5 (1372.0)	1730.3 (1657.6)	2248.4 (2179.3)	2956.0 (3106.3)
Average NIH costs per drug (no spillovers)				
Basic research on drug target^e^	1344.6 (1433.1)	1630.9 (1738.0)	2147.2 (2294.6)	2852.6 (3148.0)
Applied research on approved drug^f^	51.8 (96.8)	58.5 (111.9)	69.4 (137.8)	81.4 (168.3)
Phased development failures^g^	75.4	80.6	88.6	96.8
Estimated total NIH cost per drug (no spillovers)	1471.8	1770.0	2305.2	3030.8
Average NIH cost per drug (with spillovers)				
Basic research/drug approval (with spillovers)^h^	471.8	572.2	753.4	1000.9
Estimated total NIH cost per drug (with spillovers)	599.0	711.3	911.4	1179.1

Abbreviations: FDA, US Food and Drug Administration; NA, not applicable; NIH, National Institutes of Health; NME, new molecular entity.

^a^ All data are in millions.

^b^ Discount rates calculated on years before drug approval. The 3% and 7% discount rates are typically used to assess government investment. The 10.5% cost of capital is typically used to estimate industry costs of drug development.

^c^ The first-to-target drug is the first FDA-approved product associated with a novel biological target.

^d^ Mean (SD) NIH cost for published basic and applied research for 81 first-to-target drugs calculated after 95th percentile outlier elimination with estimated cost of phased development failures.

^e^ Mean (SD) NIH cost for published basic research on novel drug targets (n = 81) to year of first drug approval calculated after 95th percentile outlier elimination.

^f^ Mean (SD) cost for published applied research on drugs (n = 356) approved from 2010 to 2019 calculated after 95th percentile outlier elimination.

^g^ Funding from the NIH per drug for phased trials of failed drugs.

^h^ Spillovers from basic research based on 2.85 drugs associated with each biological target.

### NIH Investment in Applied Research on Drug Products

Figure 1B shows NIH-funded publications, project years, and NIH costs associated with applied research on 356 drugs through the year of approval. Before first approval, 301 of 356 products (84.5%) had NIH research funding (eTable 4 in Supplement 1). Figure 2B shows cumulative NIH costs for applied research with no discount rate or 3% and 7% discount rates. After 95th percentile outlier elimination, the mean (SD) NIH cost for applied research before approval was $51.8 ($96.8) million (3% discount, $58.5 [$111.9] million; 7% discount, $69.4 [$137.8] million; 10.5% cost of capital, $81.4 [$168.3] million) (Table 2). Outliers included searches failing to distinguish applied research on the approved product from basic research on the corresponding natural compound (ie, clotting factors, hormones, α-1 antitrypsin). Results without outlier elimination are shown in eTable 3 in Supplement 1.

### Accounting for NIH Funding for Failed Product Candidates

The NIH costs calculation associated with failed clinical trials is shown in eTable 5 in Supplement 1. Based on reported phase transition rates,^19^ 8.53 phase 1 trials, 5.08 phase 2 trials, and 1.79 phase 3 trials were conducted for each product approved. With NIH costs of $5.7 million for phase 1, $7.2 million for phase 2, and $3.9 million for phase 3,^37^ estimated NIH costs for clinical trials of failed candidates were $75.4 million for each product approval (3% discount, $80.6 million; 7% discount, $88.6 million; 10.5% cost of capital, $96.8 million) (Table 2).

### Total NIH Investment to Launch First Drug Product Associated With Novel Targets

Total NIH costs were calculated for 86 first-to-target products as the sum of NIH costs for basic research on the target, applied research on the drug, and phased clinical trials of failed compounds. The distribution of costs is shown in Figure 2C. After 95th percentile outlier elimination, mean (SD) NIH costs before a first-to-target product launch was $1.44 ($1.37) billion (3% discount, $1.73 [$1.66] billion; 7% discount, $2.24 [$2.18] billion; 10.5% cost of capital, $2.96 [$3.11] billion) (Table 2). Data without outlier elimination are shown in eTables 2 and 4 in Supplement 1.

### Comparing NIH and Industry Investments

DiMasi et al^19^ estimated average industry spending on 106 drugs approved from 1990 to 2010 at $1.5 billion or $2.8 billion with a 10.5% cost of capital (inflation-adjusted to 2018). Using different methods, Wouters et al^20^ reported an average industry spending on 63 drugs approved from 2009 to 2018 of $374.1 million, (95% CI, $301.9 million to $464.2 million) or $1.6 billion (95% CI, $1.27 billion to $1.89 billion) with a 10.5% cost of capital.

Spending from the NIH per approval for 81 first-to-target products was significantly greater than reported industry spending on 63 drugs^20^ before accounting for clinical failures, cost of capital, or discount rates (difference, −$1998.4 million; 95% CI, −$3302.1 million to −$694.6 million; *P* = .003) or with accounting for clinical failures (difference, −$1415.8 million; 95% CI, −$2731.4 million to $100.2 million; *P* = .04) (Table 3). Spending from the NIH was not less than industry spending when industry costs were estimated with clinical failures and a 10.5% cost of capital, and NIH spending was estimated with clinical failures and either a 3% discount rate (difference, −$1435.3 million; 95% CI, −$3114.6 million to $244.0 million; *P* = .09) or a 7% discount rate (difference, −$2436.3 million; 95% CI, −$4782.1 million to −$90.5 million; *P* = .04) (Table 3). Investment from the NIH and the industry was not significantly different when industry spending was estimated with clinical failures, prehuman costs^19^ (30.8% real costs), and a 10.5% cost of capital, and when NIH costs were estimated with clinical failures and either a 3% discount rate (difference, −$393.8 million; 95% CI, −$2120.5 million to $1332.9 million; *P* = .65) or 7% discount rate (difference, −$1394.8 million; 95% CI, −$3774.8 million to $985.2 million; *P* = .25) (Table 3).

**Table 3.** Comparison of the NIH Investment in Basic and Applied Research on FDA-Approved Drugs and Estimated Industry Investment in Development Accounting for Clinical Failures and the Time-Value of Investments

Include clinical failures^a^	%		Industry preclinical costs^b^	$^c^		*P* value^d^
	NIH discount rate^e^	Industry cost of capital^f^		NIH average (95% CI)^g^	Difference (NIH-industry) (95% CI)^h^	
No	0	0	Included	2372.4 (1510.1 to 3234.7)	−1998.4 (−3302.1 to −694.6)	.003
Yes	0	0	Included	2447.8 (1577.6 to 3318.0)	−1415.8 (−2731.4 to −100.2)	.04
Yes	3	10.5	Included	2994.4 (1883.7 to 4105.1)	−1435.3 (−3114.6 to 244.0)	.09
Yes	7	10.5	Included	3995.5 (2443.9 to 5547.0)	−2436.3 (−4782.1 to −90.5)	.04
Yes	3	10.5	DiMasi et al^i^	2994.4 (1852.3 to 4136.5)	−393.8 (−2120.5 to 1332.9)	.65
Yes	7	10.5	DiMasi et al^i^	3995.5 (2421.2 to 5569.7)	−1394.8 (−3774.8 to 985.2)	.25

Abbreviations: FDA, US Food and Drug Administration; NIH, National Institutes of Health; OMB, US Office of Management and Business.

^a^ Clinical failures are estimated as the average NIH costs associated with each phase of clinical development and published phase transition rates. Spending from the NIH on clinical failures was calculated using phase transition rates described by DiMasi et al.^19^ Industry spending on clinical failures was calculated using phase transition rates described by Wong et al.^23^

^b^ Investment by the NIH in preclinical research was included in applied research. Industry costs reported by Wouters et al^20^ included preclinical research with phase 1. In a separate sensitivity analysis, Wouters et al^20^ estimated preclinical costs to be 43% of clinical costs based on data reported by DiMasi et al.^19^

^c^ All data are in millions.

^d^ Univariate linear regression performed with NIH funding associated with drugs approved from 2010 to 2019 with novel drug targets (n = 81) or reported industry spending (n = 63) with an indicator variable for industry of 1 or NIH of 0.

^e^ Investment by the NIH was calculated with no discount rate or the 3% or 7% discount rates for government spending recommended by OMB.

^f^ Industry investment was calculated with a 10.5% cost of capital based on DiMasi et al^19^ and Wouters et al.^20^

^g^ Intercept of linear regression model estimates average NIH spending on basic and applied research, which included preclinical research and phased clinical trials.

^h^ Coefficient of linear regression model estimates the difference between NIH spending and industry spending. Negative values indicated NIH spending greater than industry spending.

^i^ Includes prehuman studies as 30.8% of total costs or 42.9% of costs calculated with 10.5% cost of capital as described by DiMasi et al.^19^

### Spillover Effects From Basic Research on Drug Targets

Santos et al^40^ cataloged 893 biological targets for FDA-approved products (1578) through June 2015, of which 1467 (93.0%) met inclusion criteria for this study. These products were associated with 515 biological targets, an average of 2.85 products per target (eFigure 2 in Supplement 1).

Accounting for spillovers of basic research on novel drug targets to 2.85 product approvals, the NIH cost for basic research per approval was $471.8 million (3% discount, $572.2 million; 7% discount, $753.4 million; 10.5% cost of capital, $1.0 billion) (Table 2). Accounting for spillover effects from basic research on drug targets, costs of applied research, product failures, and discount rates or cost of capital, the estimated NIH investment per approval was $599.0 million (3% discount, $711.3 million; 7% discount, $911.4 million; 10.5% cost of capital, $1179 million). Estimated NIH spending was lower than the reported average industry spending^19^ but within the 95% CI of per drug spending.^20^

## Discussion

In this cross-sectional study, evidence suggests the public sector makes substantial contributions to the foundational knowledge on which drug approvals are based,^1,2,4,6,7,8,41,42^ but less to patents^6,9^ or development.^2,3,37,43^ Conversely, the industry is primarily responsible for product development and sponsored more than 99% of the product launches in this data set.^6^

The objective of this work was to compare NIH investments in recent drug approvals with reported investment by the industry. This required an accounting for NIH spending with costs for basic research on the targets for these drugs, applied research on the approved products, phased clinical trials of failed products, and the recommended discount rates for government spending.^30,31^ This accounting adheres closely to methods used to estimate industry investment,^19,20^ while also recognizing fundamental differences in the nature of public and private sector investment in prevailing economic theories.^10^

These analyses suggest that NIH project costs for basic or applied research associated with the products approved from 2010 to 2019 were significantly greater than reported industry spending. Costs for the NIH were also higher than industry costs when both included spending on failed clinical trials of candidate products. Including clinical failures, NIH investment (calculated with either a 3% or 7% discount rate) was not less than industry investment calculated with a 10.5% cost of capital. Investment from the NIH calculated with clinical failures and a 3% or 7% discount rate was also not less than industry investment calculated with clinical failures, additional costs of prehuman research, and 10.5% cost of capital. These results suggest that NIH investments in pharmaceutical innovation are comparable with those made by industry.

While including the cost of capital in estimates of the industry’s investment in pharmaceutical innovation is controversial^44^ and estimates of this rate vary,^45,46^ consideration of the cost of capital is normative in finance theory and practice. These calculations are also consistent with prevailing economic theories that view private sector investment as inherently productive in that it typically generates a return on investment. In this context, the cost of capital represents the opportunity cost or financial risk that long-term capital investments in drug discovery and development may not achieve normal returns on investment.

There is no theoretical basis for applying an equivalent cost of capital to government spending. Prevailing economic theories treat government funding as nonproductive in that it is not expected to provide a return on investment. The 3% and 7% discount rates recommended by the US Office of Management and Budget for government spending^30,31^ have distinct theoretical foundations. The 3% discount rate on federal spending approximates the historical cost of government borrowing and, consequently, the full cost of government spending.^31,47^ The 7% discount rate represents the average productivity of private sector investments and is interpreted as a measure of the opportunity cost to the economy if public sector spending crowds out and reduces private sector investment.^30,48^ Given evidence that NIH funding for biomedical research stimulates, rather than reduces, private sector investment,^49^ estimating NIH investment with the 3% discount rate may be most consistent with prevailing economic principles.

This analysis did include an NIH spending calculation with the 10.5% cost of capital. This value provides an estimate of the additional costs that the industry would incur in the absence of NIH spending. Comparing these estimated cost savings with those of DiMasi et al^19^ or Wouters et al^20^ of industry investment suggests that industry costs would be more than double in the absence of the NIH contributions.

This work also recognizes that economic efficiencies may arise through spillover of knowledge or capabilities gained from NIH-funded basic research to applications by multiple firms or multiple products.^8,29,35,43,50^ Such spillovers would reduce the estimated NIH cost per approval. Considering only potential spillovers from NIH-funded basic research on drug targets to multiple products directed at the same targets, NIH spending per drug was within the range of actual industry spending.^20^ Spending from the NIH was estimated with either a 3% or 7% discount rate was lower than industry spending calculated with the 10.5% cost of capital.

### Policy Implications

Science and innovation policy remains grounded in a model in which government investments in basic research generate scientific capital that can be commercialized by industry for social and economic benefit. This model is exemplified by NIH spending for basic biomedical science, which plays an enabling role in pharmaceutical innovation.^1,3,6,7,28,49^ Emerging economic theory formalizes this model by contextualizing government funding for research as an early-stage investment in innovation.^10,11,12,13,15,16,17^ These theories further posit that, as early-stage investors, government or the public sector it represents could expect social or economic returns commensurate with those of comparable investments by the private sector.^10,15,16^

The present study was predicated on this concept that NIH spending represents an investment that can be meaningfully compared with investment by the industry. In this context, the finding that the magnitude of NIH investment in new drugs is comparable with that of the industry suggests that returns to the public and private sector should also be comparable. To achieve this, public policy associated with drug pricing,^51^ corporate profit,^52^ and commercial applications of government-funded invention^53^ should be calibrated to provide an equitable distribution of returns between the public and private sectors.^10,15,16^ The present results may provide a cost basis for considering not only the private rate of return to industry or the economy, but also the social return on investments,^40^ including the multiplex elements associated with health.^54,55^

### Limitations

First, this analysis is limited by the sensitivity and specificity of PubMed searches, right censoring of the data collection, and reported false-positive and false-negative associations between PMIDs and NIH projects in REPORTER.^38^ Search terms may not identify NIH funding for research tools, pharmaceutical modalities, or process development, which may underestimate total NIH costs.

Second, NIH costs for each publication were estimated as 1 fiscal year of project funding. This is consistent with evidence that 5-year NIH grants produce a median of 5 publications^56^ but may underestimate NIH costs for studies spanning multiple years.

Third, NIH funding in REPORTER represents a fraction of public sector funding for biomedical research and does not include funding from other agencies or governments, nongovernment organizations, academic institutions, or nonprofit organizations. This analysis also did not include contract funding, research and development tax credits, or vouchers. This would underestimate the public sector contribution to pharmaceutical innovation.

Fourth, this study considered only spillovers from basic research on drug targets. Spillovers may also emerge from NIH funding for research training, infrastructure, or capabilities. This would not affect the total NIH costs but would underestimate the gain from economic efficiencies.

## Conclusions

This cross-sectional study found that NIH investment in drugs approved from 2010 to 2019 was not less than investment by industry, with comparable accounting for basic and applied research, failed clinical trials, and cost of capital or discount rates. The relative scale of NIH and industry investment may provide a cost basis for calibrating the balance of social and private returns from investments in pharmaceutical innovation.

## Notes

Supplement 1. eMethods. eTable 1. PubMed drug and target search terms eTable 2. NIH costs associated with 86 novel drug targets with quartile and 95% percentile values eTable 3. NIH costs for basic and applied research related to NMEs approved 2010-2019 without outlier elimination eTable 4. NIH costs associated with 356 drugs approved 2010-2019 with quartile and 95% percentile values eTable 5. Calculation of the NIH contribution to phased clinical trials of failed clinical compounds for each product approval eFigure 1. NIH funding for basic and applied research related to drugs approved 2010-2019 by Project Activity Code eFigure 2. Number of approved FDA drugs (through June 2015) associated with 515 drug targets eReferences.

Supplement 2. Data sharing statement
