# Supplementary material for: Comparison of Research Spending on New Drug Approvals by the National Institutes of Health vs the Pharmaceutical Industry, 2010-2019
Source: JAMA Health Forum. 2023 Apr 28;4(4):e230511. doi: 10.1001/jamahealthforum.2023.0511 (PMC10148199; doi:10.1001/jamahealthforum.2023.0511)
Supplement: Supplement 1. — eMethods. eTable 1. PubMed drug and target search terms eTable 2. NIH costs associated with 86 novel drug targets with quartile and 95% percentile values eTable 3. NIH costs for basic and applied research related to NMEs approved 2010-2019 without outlier elimination eTable 4. NIH costs associated with 356 drugs approved 2010-2019 with quartile and 95% percentile values eTable 5. Calculation of the NIH contribution to phased clinical trials of failed clinical compounds for each product approval eFigure 1. NIH funding for basic and applied research related to drugs approved 2010-2019 by Project Activity Code eFigure 2. Number of approved FDA drugs (through June 2015) associated with 515 drug targets eReferences. [file jamahealthforum-e230511-s001.pdf]

Supplemental Online Content

Galkina Cleary E, Jackson MJ, Zhou EW, Ledley FD.Comparison of research spending on new drug approvals by the National Institutes of Health vs the pharmaceutical industry, 2010-2019. *JAMA Health Forum*. 2023;4(4):e230511. doi:10.1001/jamahealthforum.2023.0511

eMethods.

eTable 1. PubMed drug and target search terms

eTable 2. NIH costs associated with 86 novel drug targets with quartile and 95% percentile values

eTable 3. NIH costs for basic and applied research related to NMEs approved 2010-2019 without outlier elimination

eTable 4. NIH costs associated with 356 drugs approved 2010-2019 with quartile and 95% percentile values

eTable 5. Calculation of the NIH contribution to phased clinical trials of failed clinical compounds for each product approval

eFigure 1. NIH funding for basic and applied research related to drugs approved 2010-2019 by Project Activity Code

eFigure 2. Number of approved FDA drugs (through June 2015) associated with 515 drug targets

eReferences.

This supplemental material has been provided by the authors to give readers additional information about their work.

© 2023 Galkina Cleary E et al. *JAMA Health Forum.*

eMethods

Identifying PMID related to drugs or drug targets

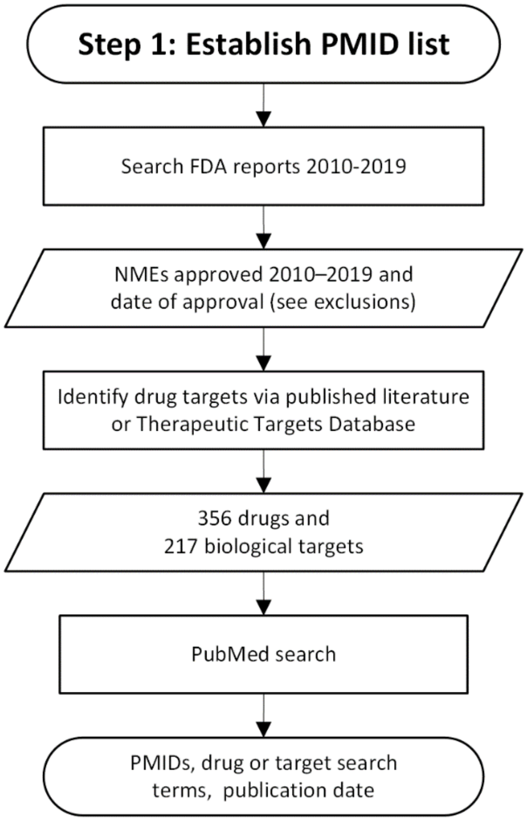

New Molecular Entities (NMEs), including both NDAs and BLAs (type 1), approved by the FDA 2010–2019 and their date of first approval were identified from FDA reports from the Center for Drug Evaluation and Research (CDER) (https://www.fda.gov/drugs/development-approval-process-drugs/new-drugs-fda-cders-new-molecular-entities-and-new-therapeutic-biological-products) and from the Center for Biologics Evaluation and Research (CBER) (https://www.fda.gov/vaccines-blood-biologics/development-approval-process-cber/2022-biological-approvals). Products derived from blood or tissue, diagnostic agents, vaccines, and antimicrobials were excluded.

Drug targets were identified for these drugs from published literature (Eder, Sedrani et al. 2014, Santos, Ursu et al. 2017) or Therapeutic Targets Database (https://db.idrblab.net/ttd/) accessed January-June 2020 (Zhu, Han et al. 2010), giving a total of 356 drugs and 217 targets.

A drug was identified as first-in-class if it was the first product associated with a novel biological target as described by (Eder, Sedrani et al. 2014) or Lanthier et al., 2011 (Lanthier, Miller et al. 2013).

PubMed searches, including MeSH terms and Boolean modifiers, were performed for each drug and target (eTable 1) (May-June 2020) using the National Center for Biological Information (NCBI) Automated Term Mapping protocols (released in March 2020) (https://www.nlm.nih.gov/pubs/techbull/ma20/ma20_pubmed_default.html). The resulting publications for each drug and target were identified by their PubMed Identifier (PMID) along with the publication date and the search term (drug ID or target ID) used in the initial search identifying that PMID. PMIDs with publication dates after first FDA drug approval were excluded.

PMIDs identified in searching for a drug name are categorized as "DRUG" and are considered applied research related to that product. PMIDs identified in searching for a drug target, but not the associated drug, are categorized as "TARGET ONLY" and are considered basic research related to products associated with that target.

© 2023 Galkina Cleary E et al. *JAMA Health Forum.*

Note: PMIDs may be identified in multiple searches and are retained in the dataset in conjunction with each search. The resulting duplication is addressed in the final step of analyzing any specific subset of drugs or drug targets (see below).

Identifying NIH funding for PMID

The NIH-funded grants (projects) and costs associated with each PMID were identified in the NIH RePORTER database (https://reporter.nih.gov/) accessed May 2020 by API (https://api.reporter.nih.gov/) and archived on a PostgreSQL server. This dataset comprised NIH-funded projects from January 2000-June 2020.

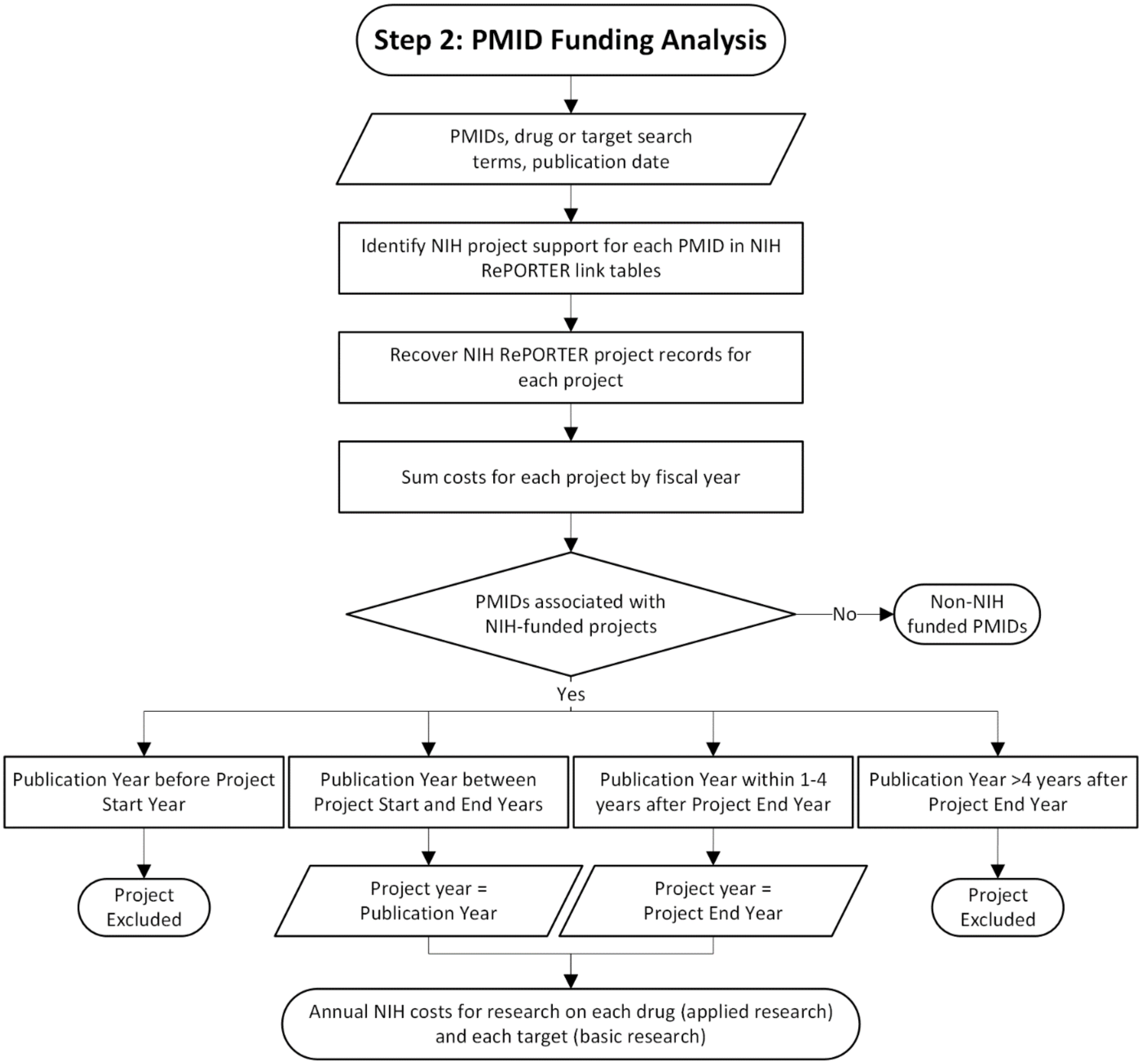

NIH RePORTER describes the funding for NIH projects, including the costs for each fiscal year, subprojects, and supplemental awards. For this analysis, each fiscal year of project funding is termed a "Project Year." All costs for each project year were totaled

© 2023 Galkina Cleary E et al. *JAMA Health Forum.*

and are termed “project year costs” or “costs.” The NIH RePORTER database also describes the project start year, project end year, and PMIDs citing funding from that award.

NIH costs associated with each PMID were identified through the following steps:

1. PMIDs were associated with one (or more) NIH-funded project cited as funding that research using the NIH RePORTER Publication “link tables”. Each project is identified by a Project Number comprising the Activity Code for that award, the institute making that award, and a unique identifier number. Data associated with each project includes the project start and end years and costs for each fiscal year, subproject, and supplemental award. Activity codes are described at (https://grants.nih.gov/grants/funding/ac_search_results.htm) and are further characterized by NIH funding program (https://grants.nih.gov/grants/funding/funding_program.htm).
2. For PMIDs with a publication date before the project start year of an associated project, no project year or NIH costs were assigned to the PMID.
3. For PMIDs with a publication date during the term of the award, the project year and project costs associated with the year of publication were assigned to the PMID.
4. For PMIDs published 1-4 years after the project end year, the project year and project costs associated with the end year were assigned to the PMID. This accounts for the estimated 3-year lag between NIH funding in RePORTER and publication dates (Boyack and Jordan 2011).
5. For PMIDs published >4 years after the end year of the project, no project years or costs were assigned.
6. Previously reported sensitivity analysis suggested that this method associates 86.3% of PMID with NIH costs, a fraction consistent with prior descriptions of false positive and negative findings in the RePORTER database (Zhou 2022).
7. Note that project years and costs may be assigned to multiple PMIDs and that each PMID may be represented more than once in the dataset (see above). The resulting duplication is addressed in the final step of analyzing any specific subset of drugs or drug targets (see below).
8. The number of PMIDs, project years, or project year costs associated with each drug were calculated from the number of PMIDs identified in the drug search, the project years associated with those PMIDs, and the costs associated with those project years after eliminating duplicates. This is categorized as applied research. Analyses were performed on all drugs and after elimination of costs beyond the 95th percentile to outliers resulting from searches contaminated with ambiguous (generic) drug names. For example, clotting factors, hormones, and proteins such as alpha-1 antitrypsin.
9. The number of PMIDs, project years, or project year costs associated with each target were calculated from the number of PMIDs identified in the target search but not the drug search, the project years associated with those PMIDs, and the

© 2023 Galkina Cleary E et al. *JAMA Health Forum.*

costs associated with those project years after eliminating duplicates. This is categorized as basic research. Analyses were performed on all drugs and after elimination of costs beyond the 95th percentile to outliers resulting from searches contaminated with ambiguous target names. For example, CD-4, bcl-2, and EGFR, which are also used as adjectives.

10. Duplicate entries arise from PMID identified in more than one search and Project Years that support multiple PMID. The PMIDs, project years, and costs associated with a subset of drugs or their targets (i.e., individual drugs or targets, first to target drugs, a therapeutic area, or Activity Codes) are determined after eliminating duplicate PMIDs, project years, and costs within that category. Within each subset a PMID is considered applied research if it is associated with a drug search within that subset and is considered basic research if it is associated only with searches for targets within that subset. A project year and its costs are considered applied research if at least one PMID supported by that project year was identified by searching for a drug in that subset and is considered basic research if every PMID supported by that project year was identified by searching for a target in that subset. Note that a PMID, project year or its costs may be represented in multiple subsets.

Analysis was performed in SQL with data in a PostgreSQL database as previously described (Cleary, Beierlein et al. 2018, Cleary, Jackson et al. 2020, Cleary and Ledley 2020). Since completion of this work, this method has been replicated in Python code that is freely available at https://github.com/BentleySciIndustry/NIH-Contribution-to-phased-clinical-development-of-drugs-approved-Supplemental-Data-Sharing.git. This method has also been described in detail at https://zenodo.org/record/7590163#.Y-LP8nbMJnI. This method can also be accessed through a dashboard at https://www.bentley.edu/centers/center-integration-science-and-industry/nih-funding-drug-innovation-dashboard

Estimating costs with discount rates

NIH costs were estimated with 3% and 7% discount rates as recommended by the Office of Management and Budget (OMB 1992, OMB 2017) as well as with a 10.5% value equivalent to the cost of capital used in estimates of industry funding by DiMasi et al. (DiMasi, Grabowski et al. 2016) and Wouters et al. (Wouters, McKee et al. 2020).

For this analysis,

1. NIH costs for research on each drug were calculated with compounded annual discount rates of 3%, 7%, or 10.5% from 2000-2020. This is categorized as applied science.
2. Per drug costs for applied research were calculated as the average of costs for applied research on all drugs in the dataset with discount rates of 3%, 7%, or 10.5%.

© 2023 Galkina Cleary E et al. *JAMA Health Forum.*

3. For first-to-target drugs, NIH costs for research on each drug target was calculated with compounded annual discount rates of 3%, 7%, or 10.5% from 2000-2020. This is categorized as basic science.
4. Per drug NIH costs for basic research were calculated as the average of costs for basic research on first-in-class drugs with discount rates of 3%, 7%, or 10.5%. This represents the average cost of basic research leading to first approval of a drug associated with that target.

Analyses were performed in Excel.

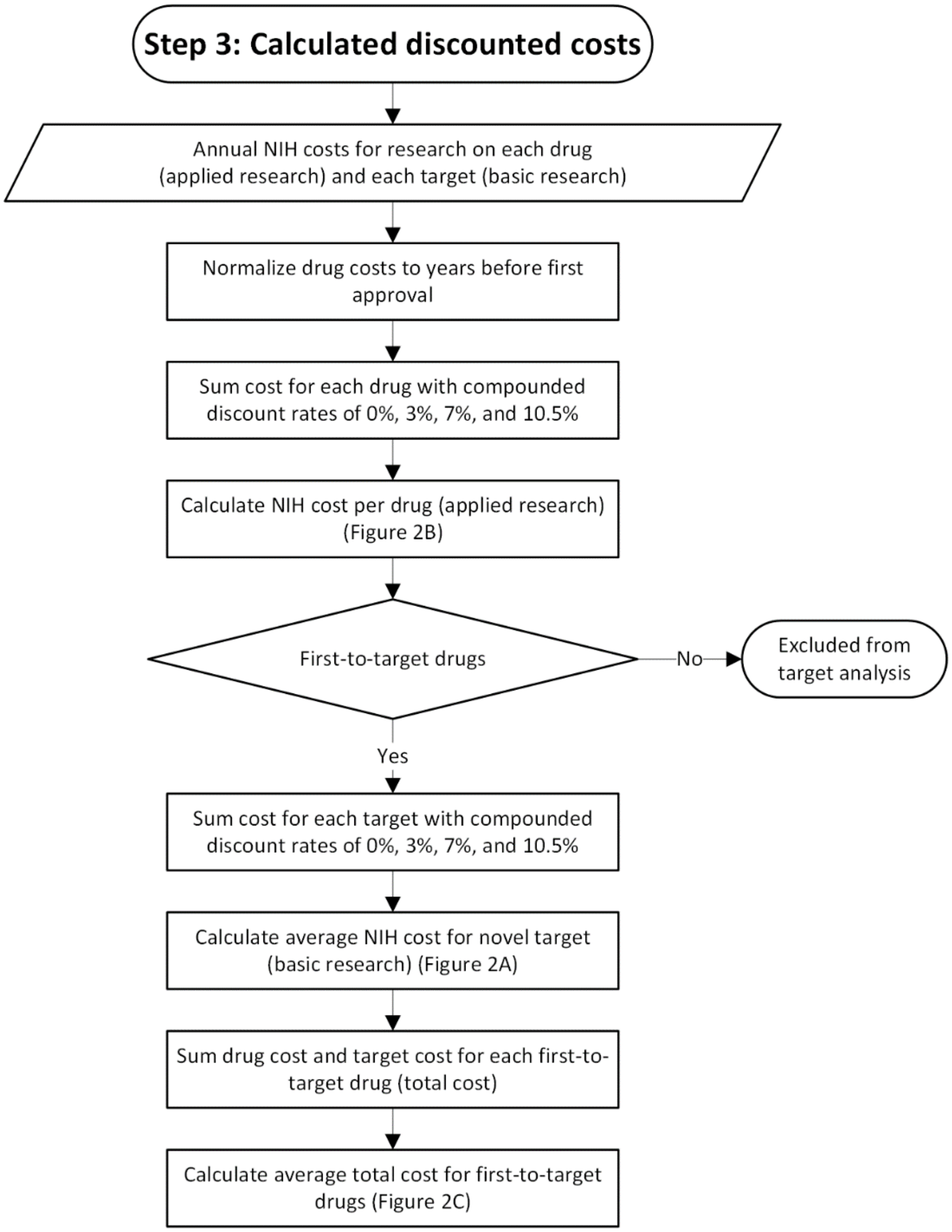

© 2023 Galkina Cleary E et al. *JAMA Health Forum.*

Estimating per drug NIH costs for failed clinical trials

Clinical phase-specific NIH funding for clinical trials of the drugs in this dataset has been described by Zhou et al. (Zhou 2022). In this analysis, PMIDs were identified by searching for the drugs in this dataset also having Publication Types “Clinical Trial,” “phase 1,” “phase 2,” or “phase 3” or having an NCT number in the abstract. PMIDs were assigned to each phase based on the Publication Type or the clinical phase associated with the NCT in clinicaltrials.gov (https://clinicaltrials.gov/ct2/home). Per drug NIH costs for each phase is the sum of the costs associated with PMIDs describing trials at that phase. Sensitivity and specificity statistics for this analysis have been described by Zhou et al. (Zhou 2022).

The clinical phase-specific success rate for products in clinical development were reported by DiMasi et al. (DiMasi, Grabowski et al. 2016). Using these fractions, the number of phase 1, phase 2, and phase 3 trials undertaken to achieve one approval was calculated. The number of failed clinical trials is the average number of trials at each phase minus 1 (eTable 5).

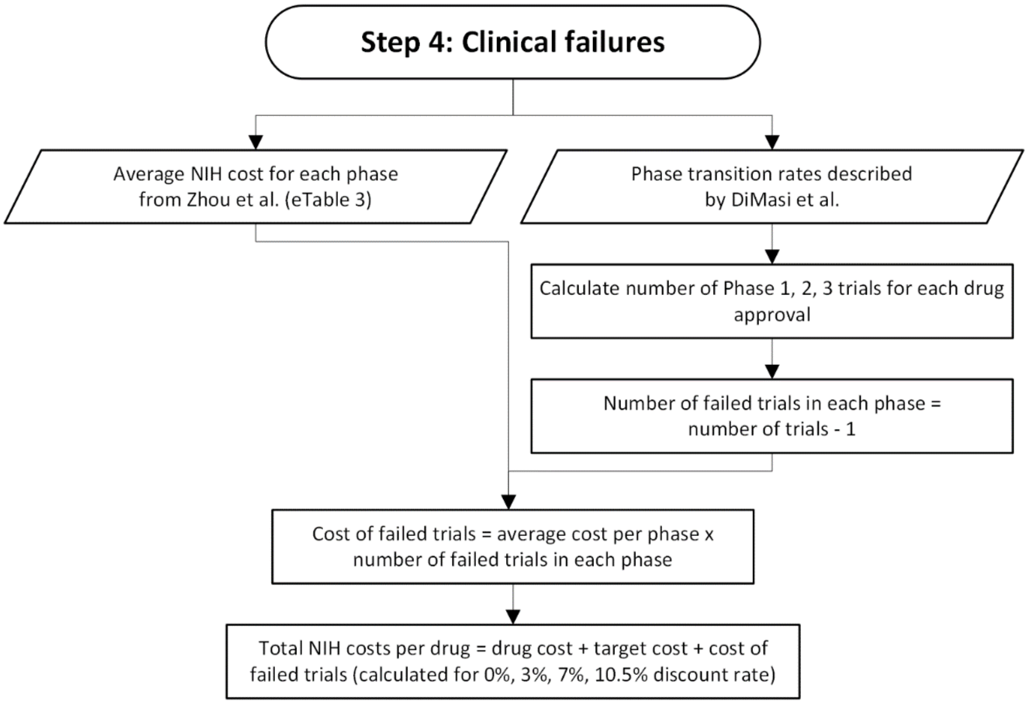

The estimated per drug cost of NIH spending for failed clinical trials is calculated as the product of the number of trials performed at each phase (not counting trials on the approved product) and the average NIH costs for each phase. It should be noted that both the clinical phase success rate described by DiMasi et al. (DiMasi, Grabowski et al. 2016) and the average NIH costs for each phase is not related to the number of individuals trials in clinicaltrials.gov, but rather the number of products proceeding through that phase and the NIH costs incurred.

© 2023 Galkina Cleary E et al. *JAMA Health Forum.*

Estimating spillover effects

Spillover effects are defined as the application of basic research on a drug target to more than one approved product. Santos et al have estimated the number of biological targets for 1,194 approved drugs. The average number of drugs/target was estimated from data in Santos et al. (Santos, Ursu et al. 2017) after eliminating drugs derived from blood or tissue, diagnostic agents, vaccines, and antimicrobials (the exclusion criteria for the present study).

NIH costs in the presence of spillover effects is estimated as total NIH costs categorized as basic research divided by the average number of drugs associated with each known biological target.

Comparison of NIH and industry costs

Average NIH costs were compared to average industry costs reported by DiMasi et al (DiMasi, Grabowski et al. 2016) or Woutres et al (2020). (Wouters, McKee et al. 2020)

Drug specific costs were compared for 81 first-in-class drugs with NIH costs estimated in the present dataset and 63 drugs with industry costs described by Wouters et al. (Wouters, McKee et al. 2020) using univariate regression,

$$Cost_i = \beta_0 + \beta_1\ Source_i$$

where $Cost_i$ is the estimated NIH or industry cost for basic and applied research on the product; and $Source_i$ is an indicator variable with a value of 0 for NIH and 1 for industry. In this model, β_0 described the median and 95% confidence interval for NIH spending and β_1 the median and 95% Confidence Interval for the difference between NIH and Industry spending.

Regression analyses were performed in Excel.

© 2023 Galkina Cleary E et al. *JAMA Health Forum.*

eTable 1. PubMed drug and target search terms. A. Drug search terms. B. Target search terms.

A. Drug search terms.

Search term ID	Brand name (Drug Search Term)	Active ingredient (Drug Search Term)	Target
drug128	Spinraza	Nusinersen	(((survival motor neuron protein) OR smn) OR smn1 smn2) OR survival of motor neuron 2 protein[MeSH Terms]
drug386	Zolgensma	onasemnogene abeparvovec-xioi	(((survival motor neuron protein) OR smn) OR smn1 smn2) OR survival of motor neuron 2 protein[MeSH Terms]
drug116	Latuda	Lurasidone	(dopamine receptor) OR serotonin receptor
drug14	Aristada	Aripiprazole lauroxil	(dopamine receptor) OR serotonin receptor
drug32	Rexulti	Brexpiprazole	(dopamine receptor) OR serotonin receptor
drug349	Caplyta	Lumateperone	(dopamine receptor) OR serotonin receptor
drug85	Addyi	Flibanserin	(dopamine receptor) OR serotonin receptor
drug198	Xuriden	Uridine triacetate	("fluorouracil"[MeSH Terms] OR ("capecitabine"[MeSH Terms]
drug186	Gattex	Teduglutide	(glucagon-like peptide 2 or glp2 or glp-2)
drug62	Unituxin	Dinutuximab	(glycolipid gd2) OR disialoganglioside gd2
drug105	Fetzima	levomilnacipran	(serotonin reuptake transporter) OR bace1
drug125	Akynzeo	Netupitant, palonosetron	(substance p receptor) OR tachykinin receptor 1
drug167	Varubi	Rolapitant	(substance p receptor) OR tachykinin receptor 1
drug264	Akynzeo IV	Palonosetron, fosnetupitant	(substance p receptor) OR tachykinin receptor 1
drug206	Viibryd	Vilazodone	5-ht1a receptors OR serotonin reuptake
drug112	Belviq	Lorcaserin	5-HT2C receptor
drug64	Northera	Droxidopa	adrenergic receptor
drug9	Lumizyme	Alglucosidase alfa	alpha glucosidase
drug200	Entyvio	Vedolizumab	alpha4beta7 integrin
drug151	Fycompa	Perampanel	AMPA 1
drug43	Zykadia	Ceritinib	anaplastic lymphoma kinase
drug48	Xalkori	Crizotinib	anaplastic lymphoma kinase
drug8	Alecensa	Alectinib	anaplastic lymphoma kinase
drug277	Erleada	Apalutamide	androgen receptor
drug329	Nubeqa	Darolutamide	androgen receptor
drug75	Xtandi	Enzalutamide	androgen receptor
drug21	Edarbi	Azilsartan	angiotensin ii type 1 receptor
drug229	Giapreza	Angiotensin II, LFPC-501	angiotensin ii type 1 receptor

© 2023 Galkina Cleary E et al. *JAMA Health Forum.*

drug121	Kynamro	Mipomersen	APOB OR apolipoprotein B
drug16	Erwinaze	Asparaginase Erwinia chrysanthemi	asparaginase[MeSH Terms]
drug204	Venclexta	Venetoclax	Bcl-2
drug158	Iclusig	Ponatinib	bcr-abl
drug122	Myrbetriq	Mirabegron	"beta 3" AND adrenergic receptor
drug182	Elelyso	Taliglucerase alfa	beta glucocerebrosidase
drug135	Striverdi Respimat	Olodaterol	beta-2-adrenergic receptor
drug197	Anoro Ellipta	Umeclidinium, vilanterol	beta-2-adrenergic receptor
drug205	Breo Ellipta	Fluticasone, vilanterol	beta-2-adrenergic receptor
drug93	Arcapta neohaler	Indacaterol	beta-2-adrenergic receptor
drug201	Vpriv	Velaglucerase alfa	"beta-glucosidase"[MeSH Terms] OR "beta-glucosidase"[All Fields]
drug92	Xeomin	IncobotulinumtoxinA	botulinum toxin A
drug89	Firazyr	Icatibant	bradykinin receptor B2
drug203	Zelboraf	Vemurafenib	braf
drug268	Braftovi	Encorafenib	braf
drug51	Tafinlar	Dabrafenib	braf
drug225	Calquence	Acalabrutinib	bruton's tyrosine kinase
drug344	Brukinsa	Zanubrutinib	bruton's tyrosine kinase
drug88	Imbruvica	Ibrutinib	bruton's tyrosine kinase
drug35	Cometriq	Cabozantinib	c-Met or hepatocyte growth factor
drug39	Carbaglu	Carglumic acid	carbamoyl-phosphate synthase
drug87	Voraxaze	Glucarpidase	carboxypeptidase G
drug131	Gazyva	Obinutuzumab	CD20
drug239	Ocrevus	Ocrelizumab	CD20
drug28	Blincyto	Blinatumomab	CD3
drug31	Adcetris	Brentuximab vedotin	CD30
drug56	Darzalex	Daratumumab	CD38
drug141	Ibrance	Palbociclib	CDK4 or CDK6
drug69	Cerdelga	Eliglustat	Ceramide glucosyltransferase OR glucosylceramide synthase
drug115	Orkambi	Ivacaftor plus lumacaftor	CFTR
drug303	Symdeko	Tezacaftor and ivacaftor	CFTR
drug299	Pifeltro	Doravirine	HIV reverse transcriptase
drug342	Trikafta	Elexacaftor, ivacaftor, tezacaftor	CFTR
drug99	Kalydeco	Ivacaftor	CFTR
drug290	Moxidectin	Moxidectin	"chloride channels"[MeSH Terms]
drug49	Fulyzaq	Crofelemer	"chloride channels"[MeSH Terms]
drug44	Cholbam	Cholic acid	cholic acid[MeSH Terms]
drug27	Zinplava	Bezlotoxumab	clostridium difficile toxin b
drug165	Xarelto	Rivaroxaban	coagulation factor X

© 2023 Galkina Cleary E et al. *JAMA Health Forum.*

drug385	Andexxa	Coagulation Factor Xa (recombinant), inactivated	coagulation factor X
drug66	Savaysa	Edoxaban	coagulation factor X
drug1	Zytiga	Abiraterone	CYP17A1
drug96	Yervoy	Ipilimumab	cytotoxic T-lymphocyte-associated protein 4 OR CTLA-4
drug90	Praxbind	Idarucizumab	Dabigatran
drug188	Aubagio	Teriflunomide	dihydroorotate dehydrogenase
drug108	Tradjenta	Linagliptin	dipeptidyl-peptidase 4
drug11	Nesina	Alogliptin	dipeptidyl-peptidase 4
drug40	Vraylar	Cariprazine	dopamine receptor
drug360	Vyondys 53	golodirsen	dystrophin
drug78	Exondys 51	Eteplirsen	dystrophin
drug124	Portrazza	Necitumumab	EGFR
drug139	Tagrisso	Osimertinib	EGFR
drug117	Opsumit	Macitentan	endothelin receptor
drug4	Gilotrif	Afatinib	erbb1 OR erbb2
drug146	Omontys	Peginesatide	erythropoietin
drug23	Sirturo	Bedaquiline	Escherichia coli ATP synthase
drug140	Osphena	Ospemifene	estrogen receptor
drug22	Duavee	Bazedoxifene acetate plus oestrogens	estrogen receptor
drug60	Natazia	Oestradiol valerate, dienogest	estrogen receptor
drug12	Eliquis	Apixaban	factor Xa
drug223	Bevyxxa	Betrixaban	factor Xa
drug129	Ocaliva	Obeticholic acid	farnesoid x receptor
drug163	Edurant	Rilpivirine	HIV reverse transcriptase
drug109	Victoza	Liraglutide	glucagon-like peptide 1
drug110	Adlyxin	Lixisenatide	glucagon-like peptide 1
drug240	Ozempic	Semaglutide	glucagon-like peptide 1
drug6	Tanzeum	Albiglutide	glucagon-like peptide 1
drug65	Trulicity	Dulaglutide	glucagon-like peptide 1
drug189	Egrifta	Tesamorelin	growth hormone-releasing hormone[MeSH Terms]
drug107	Linzess	Linaclotide	guanylyl cyclase c
drug251	Trulance	Plecanatide	guanylyl cyclase c
drug7	Lastacaft	Alcaftadine	H1 histamine receptor[MeSH Terms]
drug68	Zepatier	Elbasvir, grazoprevir	hcv ns3 OR hcv ns4a
drug175	Olysio	Simeprevir	HCV NS3
drug187	Incivek	Telaprevir	HCV NS3
drug29	Victrelis	Boceprevir	HCV NS3
drug137	Viekira Pak	Ombitasvir, paritaprevir, dasabuvir	hcv ns5a OR hcv NS3 OR HCV NS5B

© 2023 Galkina Cleary E et al. *JAMA Health Forum.*

drug102	Harvoni	Ledipasvir plus sofosbuvir	hcv ns5a
drug202	Epclusa	Sofosbuvir plus velpatasvir	hcv ns5a
drug52	Daklinza	Daclatasvir	hcv ns5a
drug176	Sovaldi	Sofosbuvir	HCV NS5B
drug207	Erivedge	Vismodegib	hedgehog signaling OR (hedgehog AND Drosophila)
drug271	Daurismo	Glasdegib	hedgehog signaling OR (hedgehog AND Drosophila)
drug152	Perjeta	Pertuzumab	HER2 OR eErb2 OR p185
drug3	Kadcyla	Ado-trastuzumab emtansine	HER2 OR eErb2 OR p185
drug142	Farydak	Panobinostat	histone deacetylases[MeSH Terms]
drug26	Beleodaq	Belinostat	histone deacetylases[MeSH Terms]
drug98	Corlanor	Ivabradine	hyperpolarization-activated cyclic nucleotide-gated channels
drug95	Tresiba	Insulin degludec	insulin receptor
drug147	Plegridy	Peginterferon beta-1A	interferon beta 1
drug101	Taltz	Ixekizumab	Interleukin 17
drug172	Cosentyx	Secukinumab	Interleukin 17
drug246	Siliq	Brodalumab	Interleukin 17
drug30	Bosulif	Bosutinib	src kinase
drug118	Nucala	Mepolizumab	Interleukin 5
drug162	Cinqair	Reslizumab	Interleukin 5
drug174	Sylvant	Siltuximab	interleukin 6 receptor
drug192	Actemra	Tocilizumab	interleukin 6 receptor
drug234	Kevzara	Sarilumab	interleukin 6 receptor
drug169	Jakafi	Ruxolitinib	janus kinases[MeSH Terms]
drug193	Xeljanz	Tofacitinib	janus kinases[MeSH Terms]
drug293	Olumiant	Baricitinib	janus kinases[MeSH Terms]
drug334	Rinvoq	Upadacitinib	janus kinases[MeSH Terms]
drug119	Myalept	Metreleptin	Leptin
drug106	Xiidra	Lifitegrast	lymphocyte function-associated antigen-1, LFA-1
drug171	Kanuma	Sebelipase alfa	lysosomal acid lipase
drug2	Tudorza Pressair	Aclidinium bromide	M3 muscarinic receptor
drug183	Hetlioz	Tasimelteon	melatonin receptor
drug46	Xiaflex	Collagenase clostridium histolyticum	"microbial collagenase"[MeSH Terms]
drug111	Juxtapid	Lomitapide	Microsomal triglyceride transfer protein
drug34	Jevtana	Cabazitaxel	microtubule assembly
drug76	Halaven	Eribulin	microtubule assembly
drug195	Mekinist	Trametinib	mitogen-activated protein kinase kinases[MeSH Terms]

© 2023 Galkina Cleary E et al. *JAMA Health Forum.*

drug288	Mektovi	Binimetinib	mitogen-activated protein kinase kinases[MeSH Terms]
drug45	Cotellic	Cobimetinib	mitogen-activated protein kinase kinases[MeSH Terms]
drug70	Vimizim	Elosulfase alfa	N Acetylgalactosamine 6 sulfatase
drug170	Entresto	Sacubitril, valsartan	Neutral Endopeptidase
drug100	Ninlaro	Ixazomib	nf-kappa b
drug61	Tecfidera	Dimethyl fumarate	nrf2 OR NFE2l2
drug180	Belsomra	Suvorexant	orexin receptor OR hypocretin receptor
drug350	Dayvigo	Lemborexant	orexin receptor OR hypocretin receptor
drug190	Brilinta	Ticagrelor	p2y receptor
drug37	Kengreal	Cangrelor	p2y receptor
drug143	Natpara	Parathyroid hormone	parathyroid hormone receptor
drug252	Tymlos	Abaloparatide	parathyroid hormone receptor
drug133	Lynparza	Olaparib	PARP
drug260	Zejula	Niraparib	PARP
drug305	Talzenna	Talazoparib	PARP
drug13	Otezla	Apremilast	phosphodiesterase 4
drug166	Daliresp	Roflumilast	phosphodiesterase 4
drug47	Eucrisa	Crisaborole	phosphodiesterase 4
drug18	Stendra	Avanafil	phosphodiesterase 5
drug132	Jetrea	Ocriplasmin	plasmin
drug168	Rubraca	Rucaparib	poly adp ribose polymerase
drug278	Firdapse	Amifampridine	potassium channel
drug54	Ampyra	Dalfampridine	potassium channel
drug81	Potiga	Ezogabine	potassium voltage-gated channel
drug196	Ella	Ulipristal	progesterone receptor[MeSH Terms]
drug265	Annovera	Segesterone acetate and ethinyl estradiol vaginal system	progesterone receptor[MeSH Terms]
drug127	Opdivo	Nivolumab	"programmed cell death 1"
drug149	Keytruda	Pembrolizumab	"programmed cell death 1"
drug283	Libtayo	Cemiplimab	"programmed cell death 1"
drug17	Tecentriq	Atezolizumab	programmed cell death-ligand 1 OR PDL1
drug219	Bavencio	Avelumab	programmed cell death-ligand 1 OR PDL1
drug232	Imfinzi	Durvalumab	programmed cell death-ligand 1 OR PDL1
drug10	Praluent	Alirocumab	proprotein convertase subtilisin kexin type 9
drug80	Repatha	Evolocumab	proprotein convertase subtilisin kexin type 9
drug181	Zioptan	Tafluprost	Prostaglandin F receptor
drug256	Vyzulta	Latanoprostene bunod	Prostaglandin F receptor
drug208	Zontivity	Vorapaxar	"protease activated receptor"

© 2023 Galkina Cleary E et al. *JAMA Health Forum.*

drug38	Kyprolis	Carfilzomib	proteasome endopeptidase complex[MeSH Terms]
drug130	Anthim	Obiltoxaximab	protective antigen anthrax
drug94	Picato	Ingenol mebutate	protein kinase c-delta[MeSH Terms]
drug103	Lenvima	Lenvatinib	receptor tyrosine kinase
drug126	Ofev	Nintedanib	receptor tyrosine kinase
drug217	Alunbrig	Brigatinib	receptor tyrosine kinase
drug245	Rydapt	Midostaurin	receptor tyrosine kinase
drug316	Xospata	Gilteritinib	receptor tyrosine kinase
drug333	Rozlytrek	Entrectinib	receptor tyrosine kinase
drug335	Inrebic	Fedratinib hydrochloride	receptor tyrosine kinase
drug178	Natroba	Spinosad	receptors, cholinergic[MeSH Terms]
drug173	Uptravi	Selexipag	receptors, epoprostenol[MeSH Terms]
drug84	Gilenya	Fingolimod	receptors, lysosphingolipid[MeSH Terms]
drug123	Movantik	Naloxegol	receptors, opioid, mu[MeSH Terms]
drug249	Symproic	Naldemedine	receptors, opioid[MeSH Terms]
drug72	Viberzi	Eluxadoline	receptors, opioid[MeSH Terms]
drug134	Lartruvo	Olaratumab	receptors, platelet-derived growth factor[MeSH Terms]
drug179	Bridion	Sugammadex	Rocuronium OR Vecuronium
drug154	Nuplazid	Pimavanserin	serotonin receptor 2a
drug209	Trintellix	Vortioxetine	serotonin receptor
drug71	Empliciti	Elotuzumab	slamf7
drug177	Odomzo	Sonidegib	smoothened
drug248	Steglatro	Ertugliflozin	sodium glucose transporter
drug36	Invokana	Canagliflozin	sodium glucose transporter
drug55	Farxiga	Dapagliflozin	sodium glucose transporter
drug74	Jardiance	Empagliflozin	sodium glucose transporter
drug164	Adempas	Riociguat	"soluble guanylate cyclase"
drug144	Signifor	Pasireotide	somatostatin
drug113	Surfaxin	Lucinactant	surfactant protein B
drug50	Pradaxa	Dabigatran	thrombin[MeSH Terms]
drug191	Lonsurf	Tipiracil plus trifluridine	thymidine phosphorylase
drug15	Strensiq	Asfotase alfa	tissue nonspecific alkaline phosphatase
drug58	Prolia	Denosumab	tnfsf11
drug25	Benlysta	Belimumab	TNFSF13B
drug148	Krystexxa	Pegloticase	urate oxidase[MeSH Terms]
drug104	Zurampic	Lesinurad	urate transporter OR SLC22A12 OR URAT1
drug161	Stivarga	Regorafenib	urea AND kinase inhibitor
drug159	Cyramza	Ramucirumab	vascular endothelial growth factor receptor-2[MeSH Terms]
drug199	Caprelsa	Vandetanib	vascular endothelial growth factor receptor-2[MeSH Terms]

© 2023 Galkina Cleary E et al. *JAMA Health Forum.*

drug150	Rapivab	Peramivir	viral neuraminidase
drug86	Horizant	Gabapentin enacarbil	voltage gated calcium channel
drug346	Xcopri	Cenobamate	voltage gated sodium channel
drug77	Aptiom	Eslicarbazepine acetate	voltage gated sodium channel
drug20	Inlyta	Axitinib	receptors, vascular endothelial growth factor[MeSH Terms]
drug210	Zaltrap	Ziv-aflibercept	receptors, vascular endothelial growth factor[MeSH Terms]
drug5	Eylea	Aflibercept	receptors, vascular endothelial growth factor[MeSH Terms]
drug120	Impavido	Miltefosine	PHENOTYPIC-NO SEARCH TERM
drug145	Veltassa	Patiromer	PHENOTYPIC-NO SEARCH TERM
drug153	Prepopik	Picosulfate	PHENOTYPIC-NO SEARCH TERM
drug155	Esbriet	Pirfenidone	PHENOTYPIC-NO SEARCH TERM
drug156	Asclera	Polidocanol	PHENOTYPIC-NO SEARCH TERM
drug157	Pomalyst	Pomalidomide	PHENOTYPIC-NO SEARCH TERM
drug194	Yondelis	Trabectedin	PHENOTYPIC-NO SEARCH TERM
drug211	Ferriprox	Deferiprone	PHENOTYPIC-NO SEARCH TERM
drug242	Prevymis	Letermovir	PHENOTYPIC-NO SEARCH TERM
drug243	Radicava	Edaravone	PHENOTYPIC-NO SEARCH TERM
drug276	Epidiolex	cannabidiol	PHENOTYPIC-NO SEARCH TERM
drug282	Krintafel	Tafenoquine	PHENOTYPIC-NO SEARCH TERM
drug302	Seysara	Sarecycline	PHENOTYPIC-NO SEARCH TERM
drug33	Briviact	Brivaracetam	PHENOTYPIC-NO SEARCH TERM
drug332	Pretomanid	Pretomanid	PHENOTYPIC-NO SEARCH TERM
drug53	Dalvance	Dalbavancin	PHENOTYPIC-NO SEARCH TERM
drug57	Defitelio	Defibrotide sodium	PHENOTYPIC-NO SEARCH TERM
drug59	Kybella	Deoxycholic acid	PHENOTYPIC-NO SEARCH TERM
drug79	Vascepa	Ethyl eicosapentaenoic acid	PHENOTYPIC-NO SEARCH TERM
drug215	Zinbryta	Daclizumab	interleukin 2 receptor
drug222	Besponsa	Inotuzumab ozogamicin	CD22
drug287	Lumoxiti	Moxetumomab pasudotox	CD22
drug224	Brineura	Cerliponase alfa	Tripeptidyl-peptidase
drug250	Tremfya	Guselkumab	interleukin 23
drug281	Ilumya	Tildrakizumab	interleukin 23
drug355	Skyrizi	risankizumab-rzaa	interleukin 23
drug237	Mepsevii	Vestronidase alfa-vjbk	glucuronidase

© 2023 Galkina Cleary E et al. *JAMA Health Forum.*

drug228	Fasenra	Benralizumab	interleukin 5 receptor
drug255	Vosevi	Sofosbuvir, velpatasvir, voxilaprevir	Hepatitis C virus protease
drug216	Aliqopa	Copanlisib dihydrochloride	Phosphoinositide 3-kinase OR PI-3 kinase
drug269	Copiktra	Duvelisib	Phosphoinositide 3-kinase OR PI-3 kinase
drug325	Piqray	Alpelisib	Phosphoinositide 3-kinase OR PI-3 kinase
drug91	Zydelig	Idelalisib	Phosphoinositide 3-kinase OR PI-3 kinase
drug231	Idhifa	Enasidenib mesylate	isocitrate dehydrogenase
drug308	Tibsovo	Ivosidenib	isocitrate dehydrogenase
drug235	Kisqali	Ribociclib succinate	"cyclin-dependent kinase"
drug254	Verzenio	Abemaciclib	"cyclin-dependent kinase"
drug218	Austedo	Deutetrabenazine	monoamine transporter
drug233	Ingrezza	Valbenazine tosylate	monoamine transporter
drug257	Xadago	Safinamide	monoamine oxidase B
drug259	Xermelo	Telotristat etiprate	Tryptophan hydroxylase
drug295	Onpattro	Patisiran	Transthyretin
drug241	Parsabiv	Etelcalcetide	"calcium sensing receptor"
drug227	Emflaza	Deflazacort	glucocorticoid receptor OR NR3C1
drug244	Rhopressa	Netarsudil	Rho-associated protein kinase
drug310	Trogarzo	Ibalizumab	CD4
drug266	Asparlas	Calaspargase pegol	"asparagine"[MeSH Terms]
drug274	Elzonris	Tagraxofusp	CD123
drug300	Poteligeo	Mogamulizumab	CCR4
drug304	Takhzyro	Lanadelumab	plasma kallikrein
drug262	Aimovig	Erenumab	calcitonin gene-related peptide receptor
drug351	Ubrelvy	Ubrogepant	calcitonin gene-related peptide receptor
drug263	Ajovy	Fremanezumab	calcitonin gene-related peptide
drug275	Emgality	Galcanezumab	calcitonin gene-related peptide
drug280	Gamifant	Emapalumab	interferon gamma
drug311	Ultomiris	Ravulizumab	"complement C5 protein"
drug270	Crysvita	Burosumab	Fibroblast growth factor 23 OR FGF23
drug298	Palynziq	Pegvaliase	phenylalanine ammonia
drug267	Biktarvy	Bictegravir, emtricitabine, tenofovir alafenamide	HIV integrase
drug63	Tivicay	Dolutegravir	HIV integrase
drug309	TPOXX	Tecovirimat	"orthopoxvirus envelope"
drug285	Lorbrena	Lorlatinib	ALK tyrosine kinase receptor

© 2023 Galkina Cleary E et al. *JAMA Health Forum.*

drug312	Vitrakvi	Larotrectinib	tropomyosin receptor kinases
drug238	Nerlynx	Neratinib maleate	Epidermal growth factor receptor
drug313	Vizimpro	Dacomitinib	Epidermal growth factor receptor
drug273	Doptelet	Avatrombopag	Thrombopoietin receptor
drug291	Mulpleta	Lusutrombopag	Thrombopoietin receptor
drug306	Tavalisse	Fostamatinib	spleen tyrosine kinase
drug212	Onfi	Clobazam	GABA-A receptor
drug272	Diacomit	Stiripentol	GABA-A receptor
drug320	Zulresso	Brexanolone	GABA-A receptor
drug286	Lucemyra	Lofexidine	alpha-2-adrenergic receptor
drug307	Tegsedi	Inotersen	Transthyretin
drug324	Vyndaqel	Tafamidis meglumine	Transthyretin
drug296	Orilissa	Elagolix sodium	Gonadotropin-releasing hormone receptor
drug279	Galafold	Migalastat	alpha-galactosidase
drug289	Motegrity	Prucalopride	5-HT4
drug354	Evenity	Romosozumab	sclerostin
drug356	Polivy	Polatuzumab vedotin-PIIQ	CD79b
drug358	Reblozyl	Luspatercept-AAMT	tgf beta
drug361	Padcev	Enfortumab vedotin-EJFV	Nectin
drug352	Cablivi	caplacizumab-yhdp	von willebrand factor
drug376	Vonvendi	von Willebrand factor (Recombinant)	von willebrand factor
drug359	Adakveo	Crizanlizumab-TMCA	p-selectin
drug353	Jeuveau	Prabotulinumtoxina-XVFS	SNAP25 OR SNAP-25
drug357	Beovu	Brolucizumab-DBII	vascular endothelial growth factor A
drug323	Balversa	Erdafitinib	fibroblast growth factor receptor
drug327	Xpovio	Selinexor	exportin
drug347	Oxbryta	Voxelotor	hemoglobin S
drug322	Mayzent	Siponimod	sphingosine 1-phosphate
drug326	Vyleesi	Bremelanotide	melanocortin receptor
drug340	Scenesse	Afamelanotide	melanocortin receptor
drug331	Wakix	Pitolisant hydrochloride	histamine H3 receptor
drug338	Ibsrela	Tenapanor	sodium hydrogen exchanger
drug337	Nourianz	Istradefylline	"adenosine receptor"
drug339	Aklief	Trifarotene	"retinoic acid receptor"
drug345	Givlaari	Givosiran sodium	aminolevulinate synthase
drug363	Provenge	Sipuleucel-T	Prostatic acid phosphatase
drug364	Glassia	Alpha-1-proteinase inhibitor	Alpha-1-antitrypsin
drug365	Novoeight	Turoctocog alfa	coagulation "factor VIII"
drug368	Obizur	Susoctocog alfa	coagulation "factor VIII"

© 2023 Galkina Cleary E et al. *JAMA Health Forum*.

drug370	Eloctate	Efmoroctocog alfa	coagulation "factor VIII"
drug372	Adynovate	Antihemophilic Factor (Recombinant), PEGylated	coagulation "factor VIII"
drug374	Nuwiq	Simoctocog alfa	coagulation "factor VIII"
drug378	Kovaltry	Recombinant Antihemophilic Factor VIII (Human)	coagulation "factor VIII"
drug379	Afstyla	Lonoctocog alfa	coagulation "factor VIII"
drug384	Jivi	Damoctocog alfa pegol	coagulation "factor VIII"
drug387	Esperoct	Antihemophilic Factor (recombinant), GlycoPEGylated-exei	coagulation "factor VIII"
drug366	Rixubis	Coagulation Factor IX (Recombinant)	coagulation factor IX
drug371	Alprolix	Eftrenonacog alfa	coagulation factor IX
drug373	Ixinity	Recombinant Coagulation factor IX	coagulation factor IX
drug377	Idelvion	Albutrepenonacog alfa	coagulation factor IX
drug380	Rebinyn	Nonacog beta<br><br>pegol	coagulation factor IX
drug367	Tretten	Coagulation Factor XIII A Subunit (Recombinant)	Coagulation Factor XIII
drug369	Ruconest	Conestat alfa	C1 esterase
drug213	Neutroval	Tbo-Filgrastim	Granulocyte-macrophage colony-stimulating factor
drug375	Imlygic	Talimogene laherparepvec	Granulocyte-macrophage colony-stimulating factor
drug381	Kymriah	Tisagenlecleucel	CD19
drug382	Yescarta	Axicabtagene ciloleucel	CD19
drug383	Luxturna	Voretigene neparvovec	retinoid isomerohydrolase OR RPE65
drug297	Oxervate	Cenegermin	"nerve growth factor"
drug315	Xofluza	Baloxavir marboxil	Polymerase Acidic Endonuclease
drug236	Mavyret	Glecaprevir, pibrentasvir	NS3 protease OR Nonstructural protein 5A
drug317	Yupelri	Revefenacin	muscarinic receptor
drug226	Dupixent	Dupilumab	interleukin 4 receptor
drug73	Genvoya	Elvitegravir, cobicistat, emtricitabine, tenofovir	HIV integrase or CYP3A

© 2023 Galkina Cleary E et al. *JAMA Health Forum.*

drug362	Enhertu	Fam-Trastuzumab Deruxtecan-NXKI	HER2 OR eErb2 OR p185 or topoisomerase i
drug230	Hemlibra	Emicizumab	Coagulation factor IX OR Coagulation factor X
drug24	Nulojix	Belatacept	CD80 OR CD86
drug330	Turalio	Pexidartinib hydrochloride	cd117 OR cd115
drug341	Reyvow	Lasmiditan succinate	5-HT1 receptor
drug321	Sunosi	Solriamfetol	(noradrenaline reuptake) OR (dopamine reuptake)

© 2023 Galkina Cleary E et al. *JAMA Health Forum.*

B. Target search terms.

Search term ID	Brand name (Drug Search Term)	Active ingredient (Drug Search Term)	Target
2	(null)	(null)	(((survival motor neuron protein) OR smn) OR smn1 smn2) OR survival of motor neuron 2 protein[MeSH Terms]
3	(null)	(null)	(dopamine receptor) OR serotonin receptor
4	(null)	(null)	("fluorouracil"[MeSH Terms] OR ("capecitabine"[MeSH Terms]
5	(null)	(null)	(glucagon-like peptide 2 or glp2 or glp-2)
6	(null)	(null)	(glycolipid gd2) OR disialoganglioside gd2
7	(null)	(null)	(serotonin reuptake transporter) OR bace1
8	(null)	(null)	(substance p receptor) OR tachykinin receptor 1
9	(null)	(null)	5-ht1a receptors OR serotonin reuptake
10	(null)	(null)	5-HT2C receptor
11	(null)	(null)	adrenergic receptor
13	(null)	(null)	alpha glucosidase
14	(null)	(null)	alpha4beta7 integrin
15	(null)	(null)	AMPA 1
16	(null)	(null)	anaplastic lymphoma kinase
17	(null)	(null)	androgen receptor
18	(null)	(null)	angiotensin ii type 1 receptor
20	(null)	(null)	APOB OR apolipoprotein B
21	(null)	(null)	asparaginase[MeSH Terms]
23	(null)	(null)	Bcl-2
24	(null)	(null)	bcr-abl
25	(null)	(null)	"beta 3" AND adrenergic receptor
26	(null)	(null)	beta glucocerebrosidase
28	(null)	(null)	beta-2-adrenergic receptor
29	(null)	(null)	"beta-glucosidase"[MeSH Terms] OR "beta-glucosidase"[All Fields]
30	(null)	(null)	botulinum toxin A
31	(null)	(null)	bradykinin receptor B2
32	(null)	(null)	braf
33	(null)	(null)	bruton's tyrosine kinase
34	(null)	(null)	c-Met or hepatocyte growth factor
35	(null)	(null)	carbamoyl-phosphate synthase
36	(null)	(null)	carboxypeptidase G
37	(null)	(null)	CD20
38	(null)	(null)	CD3
39	(null)	(null)	CD30
40	(null)	(null)	CD38

© 2023 Galkina Cleary E et al. *JAMA Health Forum.*

41	(null)	(null)	CDK4 or CDK6
43	(null)	(null)	Ceramide glucosyltransferase OR glucosylceramide synthase
44	(null)	(null)	CFTR
66	(null)	(null)	HIV reverse transcriptase
45	(null)	(null)	"chloride channels"[MeSH Terms]
46	(null)	(null)	cholic acid[MeSH Terms]
47	(null)	(null)	clostridium difficile toxin b
48	(null)	(null)	coagulation factor X
49	(null)	(null)	CYP17A1
50	(null)	(null)	cytotoxic T-lymphocyte-associated protein 4 OR CTLA-4
51	(null)	(null)	Dabigatran
52	(null)	(null)	dihydroorotate dehydrogenase
53	(null)	(null)	dipeptidyl-peptidase 4
55	(null)	(null)	dopamine receptor
56	(null)	(null)	dystrophin
57	(null)	(null)	EGFR
58	(null)	(null)	endothelin receptor
59	(null)	(null)	erbb1 OR erbb2
60	(null)	(null)	erythropoietin
61	(null)	(null)	Escherichia coli ATP synthase
62	(null)	(null)	estrogen receptor
63	(null)	(null)	factor Xa
64	(null)	(null)	farnesoid x receptor
67	(null)	(null)	glucagon-like peptide 1
68	(null)	(null)	growth hormone-releasing hormone[MeSH Terms]
69	(null)	(null)	guanylyl cyclase c
70	(null)	(null)	H1 histamine receptor[MeSH Terms]
71	(null)	(null)	hcv ns3 OR hcv ns4a
72	(null)	(null)	HCV NS3
73	(null)	(null)	hcv ns5a OR hcv NS3 OR HCV NS5B
74	(null)	(null)	hcv ns5a
75	(null)	(null)	HCV NS5B
76	(null)	(null)	hedgehog signaling OR (hedgehog AND Drosophila)
77	(null)	(null)	HER2 OR eErb2 OR p185
78	(null)	(null)	histone deacetylases[MeSH Terms]
79	(null)	(null)	hyperpolarization-activated cyclic nucleotide-gated channels
80	(null)	(null)	insulin receptor
81	(null)	(null)	interferon beta 1
82	(null)	(null)	Interleukin 17
138	(null)	(null)	src kinase

© 2023 Galkina Cleary E et al. *JAMA Health Forum.*

83	(null)	(null)	Interleukin 5
84	(null)	(null)	interleukin 6 receptor
85	(null)	(null)	janus kinases[MeSH Terms]
86	(null)	(null)	Leptin
88	(null)	(null)	lymphocyte function-associated antigen-1, LFA-1
89	(null)	(null)	lysosomal acid lipase
90	(null)	(null)	M3 muscarinic receptor
91	(null)	(null)	melatonin receptor
92	(null)	(null)	"microbial collagenase"[MeSH Terms]
93	(null)	(null)	Microsomal triglyceride transfer protein
94	(null)	(null)	microtubule assembly
96	(null)	(null)	mitogen-activated protein kinase kinases[MeSH Terms]
97	(null)	(null)	N Acetylgalactosamine 6 sulfatase
98	(null)	(null)	Neutral Endopeptidase
99	(null)	(null)	nf-kappa b
100	(null)	(null)	nrf2 OR NFE2l2
101	(null)	(null)	orexin receptor OR hypocretin receptor
103	(null)	(null)	p2y receptor
104	(null)	(null)	parathyroid hormone receptor
105	(null)	(null)	PARP
107	(null)	(null)	phosphodiesterase 4
108	(null)	(null)	phosphodiesterase 5
110	(null)	(null)	plasmin
111	(null)	(null)	poly adp ribose polymerase
112	(null)	(null)	potassium channel
113	(null)	(null)	potassium voltage-gated channel
114	(null)	(null)	progesterone receptor[MeSH Terms]
115	(null)	(null)	"programmed cell death 1"
116	(null)	(null)	programmed cell death-ligand 1 OR PDL1
117	(null)	(null)	proprotein convertase subtilisin kexin type 9
118	(null)	(null)	Prostaglandin F receptor
119	(null)	(null)	"protease activated receptor"
120	(null)	(null)	proteasome endopeptidase complex[MeSH Terms]
121	(null)	(null)	protective antigen anthrax
122	(null)	(null)	protein kinase c-delta[MeSH Terms]
123	(null)	(null)	receptor tyrosine kinase
124	(null)	(null)	receptors, cholinergic[MeSH Terms]
125	(null)	(null)	receptors, epoprostenol[MeSH Terms]
126	(null)	(null)	receptors, lysosphingolipid[MeSH Terms]
127	(null)	(null)	receptors, opioid, mu[MeSH Terms]

© 2023 Galkina Cleary E et al. *JAMA Health Forum.*

128	(null)	(null)	receptors, opioid[MeSH Terms]
129	(null)	(null)	receptors, platelet-derived growth factor[MeSH Terms]
130	(null)	(null)	Rocuronium OR Vecuronium
131	(null)	(null)	serotonin receptor 2a
132	(null)	(null)	serotonin receptor
133	(null)	(null)	slamf7
134	(null)	(null)	smoothened
135	(null)	(null)	sodium glucose transporter
136	(null)	(null)	"soluble guanylate cyclase"
137	(null)	(null)	somatostatin
140	(null)	(null)	surfactant protein B
141	(null)	(null)	thrombin[MeSH Terms]
142	(null)	(null)	thymidine phosphorylase
143	(null)	(null)	tissue nonspecific alkaline phosphatase
144	(null)	(null)	tnfsf11
145	(null)	(null)	TNFSF13B
146	(null)	(null)	urate oxidase[MeSH Terms]
147	(null)	(null)	urate transporter OR SLC22A12 OR URAT1
148	(null)	(null)	urea AND kinase inhibitor
149	(null)	(null)	vascular endothelial growth factor receptor-2[MeSH Terms]
150	(null)	(null)	viral neuraminidase
151	(null)	(null)	voltage gated calcium channel
152	(null)	(null)	voltage gated sodium channel
153	(null)	(null)	receptors, vascular endothelial growth factor[MeSH Terms]
154	(null)	(null)	PHENOTYPIC-NO SEARCH TERM
155	(null)	(null)	interleukin 2 receptor
156	(null)	(null)	CD22
157	(null)	(null)	Tripeptidyl-peptidase
158	(null)	(null)	interleukin 23
159	(null)	(null)	glucuronidase
160	(null)	(null)	interleukin 5 receptor
161	(null)	(null)	Hepatitis C virus protease
109	(null)	(null)	Phosphoinositide 3-kinase OR PI-3 kinase
163	(null)	(null)	isocitrate dehydrogenase
164	(null)	(null)	"cyclin-dependent kinase"
165	(null)	(null)	monoamine transporter
166	(null)	(null)	monoamine oxidase B
167	(null)	(null)	Tryptophan hydroxylase
192	(null)	(null)	Transthyretin
168	(null)	(null)	"calcium sensing receptor"

© 2023 Galkina Cleary E et al. *JAMA Health Forum.*

169	(null)	(null)	glucocorticoid receptor OR NR3C1
170	(null)	(null)	Rho-associated protein kinase
172	(null)	(null)	CD4
173	(null)	(null)	"asparagine"[MeSH Terms]
174	(null)	(null)	CD123
175	(null)	(null)	CCR4
176	(null)	(null)	plasma kallikrein
177	(null)	(null)	calcitonin gene-related peptide receptor
178	(null)	(null)	calcitonin gene-related peptide
179	(null)	(null)	interferon gamma
180	(null)	(null)	"complement C5 protein"
181	(null)	(null)	Fibroblast growth factor 23 OR FGF23
182	(null)	(null)	phenylalanine ammonia
183	(null)	(null)	HIV integrase
184	(null)	(null)	"orthopoxvirus envelope"
185	(null)	(null)	ALK tyrosine kinase receptor
186	(null)	(null)	tropomyosin receptor kinases
187	(null)	(null)	Epidermal growth factor receptor
188	(null)	(null)	Thrombopoietin receptor
189	(null)	(null)	spleen tyrosine kinase
190	(null)	(null)	GABA-A receptor
191	(null)	(null)	alpha-2-adrenergic receptor
193	(null)	(null)	Gonadotropin-releasing hormone receptor
194	(null)	(null)	alpha-galactosidase
195	(null)	(null)	5-HT4
196	(null)	(null)	sclerostin
197	(null)	(null)	CD79b
198	(null)	(null)	tgf beta
199	(null)	(null)	Nectin
200	(null)	(null)	von willebrand factor
201	(null)	(null)	p-selectin
202	(null)	(null)	SNAP25 OR SNAP-25
203	(null)	(null)	vascular endothelial growth factor A
204	(null)	(null)	fibroblast growth factor receptor
205	(null)	(null)	exportin
206	(null)	(null)	hemoglobin S
207	(null)	(null)	sphingosine 1-phosphate
208	(null)	(null)	melanocortin receptor
209	(null)	(null)	histamine H3 receptor
210	(null)	(null)	sodium hydrogen exchanger
211	(null)	(null)	"adenosine receptor"
212	(null)	(null)	"retinoic acid receptor"

© 2023 Galkina Cleary E et al. *JAMA Health Forum.*

213	(null)	(null)	aminolevulinate synthase
214	(null)	(null)	Prostatic acid phosphatase
215	(null)	(null)	Alpha-1-antitrypsin
216	(null)	(null)	coagulation "factor VIII"
217	(null)	(null)	coagulation factor IX
218	(null)	(null)	Coagulation Factor XIII
219	(null)	(null)	C1 esterase
220	(null)	(null)	Granulocyte-macrophage colony-stimulating factor
221	(null)	(null)	CD19
222	(null)	(null)	retinoid isomerohydrolase OR RPE65
223	(null)	(null)	"nerve growth factor"
224	(null)	(null)	Polymerase Acidic Endonuclease
225	(null)	(null)	NS3 protease OR Nonstructural protein 5A
226	(null)	(null)	muscarinic receptor
227	(null)	(null)	interleukin 4 receptor
228	(null)	(null)	HIV integrase or CYP3A
229	(null)	(null)	HER2 OR eErb2 OR p185 or topoisomerase i
230	(null)	(null)	Coagulation factor IX OR Coagulation factor X
231	(null)	(null)	CD80 OR CD86
232	(null)	(null)	cd117 OR cd115
233	(null)	(null)	5-HT1 receptor
234	(null)	(null)	(noradrenaline reuptake) OR (dopamine reuptake)

© 2023 Galkina Cleary E et al. *JAMA Health Forum.*

eTable 2. NIH costs associated with 86 novel drug targets with quartile and 95% percentile values

Percentile	0%	3%	7%	10.5%
25%	$358.8	$445.0	$556.9	$668.3
50%	$827.6	$1,005.4	$1,339.6	$1,664.6
75%	$2,274.4	$2,721.5	$3,462.7	$4,651.2
95%	$7,373.0	$9,120.4	$12,292.0	$15,970.0
**Drug Targets**	**0%**	**3%**	**7%**	**10.5%**
CD4	$41,750.9	$53,792.9	$77,077.6	$104,377.6
EGFR	$17,477.5	$20,969.1	$27,072.8	$32,063.2
Phosphoinositide 3-kinase OR PI-3 kinase	$13,847.6	$16,769.1	$21,905.9	$27,215.5
Bcl-2	$8,930.0	$11,343.6	$15,863.0	$21,153.7
CD3	$7,200.0	$8,873.4	$11,895.2	$15,394.0
Leptin	$7,098.1	$8,604.1	$11,267.8	$14,216.8
Interleukin 17	$4,054.6	$4,679.7	$5,677.9	$6,709.5
mitogen-activated protein kinase kinases[MeSH Terms]	$3,967.8	$4,788.2	$6,226.8	$7,786.3
Granulocyte-macrophage colony-stimulating factor	$3,782.7	$4,552.1	$5,889.3	$11,662.3
CD19	$3,588.2	$4,444.5	$6,060.4	$7,969.1
CD38	$3,519.8	$4,243.4	$5,548.5	$7,121.2
PARP	$3,178.1	$3,716.8	$4,640.6	$5,473.9
p-selectin	$3,036.8	$3,992.4	$5,861.4	$8,070.7
c-Met or hepatocyte growth factor	$2,925.3	$3,426.6	$4,276.5	$5,160.6
receptors, vascular endothelial growth factor[MeSH Terms]	$2,741.4	$3,147.8	$3,812.3	$5,379.1
interleukin 4 receptor	$2,703.2	$3,478.1	$4,981.4	$6,945.9
hedgehog signaling OR (hedgehog AND Drosophila)	$2,547.5	$2,910.8	$3,502.2	$4,059.1
von willebrand factor	$2,517.9	$3,099.2	$4,147.7	$8,773.3
CDK4 or CDK6	$2,400.7	$2,940.3	$3,934.6	$5,034.7
interleukin 6 receptor	$2,383.5	$2,752.9	$3,359.6	$3,994.9
isocitrate dehydrogenase	$2,313.4	$2,690.1	$3,307.9	$3,965.4
"programmed cell death 1"	$2,298.7	$2,577.9	$3,014.7	$3,299.7
melanocortin receptor	$2,250.2	$2,895.2	$4,125.7	$5,708.2
janus kinases[MeSH Terms]	$2,202.6	$2,526.9	$3,066.9	$3,568.6

© 2023 Galkina Cleary E et al. *JAMA Health Forum.*

(((survival motor neuron protein) OR smn) OR smn1 smn2) OR survival of motor neuron 2 protein[MeSH Terms]	$2,121.0	$2,556.9	$3,329.5	$6,329.9
hemoglobin S	$2,109.2	$2,768.4	$4,088.5	$5,851.7
Fibroblast growth factor 23 OR FGF23	$2,107.3	$2,504.9	$3,170.8	$3,170.7
spleen tyrosine kinase	$2,101.4	$2,662.8	$3,732.3	$4,971.7
CFTR	$2,041.6	$2,362.5	$2,901.7	$3,333.0
braf	$1,910.0	$2,101.9	$2,394.6	$2,600.9
exportin	$1,798.2	$2,288.2	$3,206.8	$4,330.7
sodium hydrogen exchanger	$1,595.3	$2,189.9	$3,423.1	$5,036.3
glucuronidase	$1,556.0	$1,931.6	$2,621.3	$3,375.5
cytotoxic T-lymphocyte-associated protein 4 OR CTLA-4	$1,513.8	$1,766.6	$2,193.8	$2,638.9
dystrophin	$1,393.8	$1,698.4	$2,248.9	$2,889.7
Interleukin 5	$1,130.7	$1,401.5	$1,904.1	$2,495.8
coagulation factor X	$1,053.8	$1,259.1	$1,613.6	$2,006.5
"orthopoxvirus envelope"	$970.3	$1,239.8	$1,744.5	$2,383.1
CD22	$951.4	$1,212.5	$1,721.6	$2,279.6
Tryptophan hydroxylase	$923.1	$1,176.5	$1,647.6	$2,231.8
"protease activated receptor"	$909.0	$1,088.2	$1,396.6	$1,658.5
lymphocyte function-associated antigen-1, LFA-1	$886.9	$1,178.1	$1,758.6	$2,540.9
farnesoid x receptor	$852.3	$1,027.0	$1,339.6	$1,557.6
alpha4beta7 integrin	$827.6	$953.7	$1,163.5	$1,398.7
plasmin	$824.4	$1,005.4	$1,320.8	$1,664.6
CCR4	$779.0	$967.2	$1,324.9	$1,770.9
CD123	$736.8	$979.7	$1,482.0	$2,164.2
sclerostin	$680.5	$815.9	$1,041.6	$1,197.8
tropomyosin receptor kinases	$636.5	$760.0	$978.0	$1,219.0
calcitonin gene-related peptide receptor	$631.3	$821.2	$1,185.4	$1,645.5
Nectin	$538.6	$677.7	$936.5	$1,228.6
orexin receptor OR hypocretin receptor	$530.2	$647.5	$854.1	$1,098.8
retinoid isomerohydrolase OR RPE65	$527.0	$660.4	$903.0	$1,197.9
tnfsf11	$485.3	$569.9	$708.6	$839.8
hcv ns5a	$469.6	$552.3	$696.8	$854.4
bruton's tyrosine kinase	$460.7	$534.7	$662.3	$783.0

© 2023 Galkina Cleary E et al. *JAMA Health Forum.*

"soluble guanylate cyclase"	$443.3	$570.0	$809.5	$1,110.0
CD30	$439.5	$507.9	$620.3	$743.7
interleukin 5 receptor	$416.2	$525.3	$737.7	$1,020.3
anaplastic lymphoma kinase	$410.5	$460.4	$540.6	$600.9
(glycolipid gd2) OR disialoganglioside gd2	$407.8	$477.6	$592.6	$644.8
TNFSF13B	$400.9	$464.2	$567.9	$677.9
hyperpolarization-activated cyclic nucleotide-gated channels	$371.6	$443.9	$568.4	$710.0
HCV NS3	$363.0	$446.2	$594.7	$765.8
CD79b	$354.5	$454.2	$649.5	$909.4
lysosomal acid lipase	$346.1	$418.6	$545.9	$688.4
bradykinin receptor B2	$291.0	$344.3	$434.4	$504.0
clostridium difficile toxin b	$276.0	$336.4	$448.0	$578.9
receptors, lysosphingolipid[MeSH Terms]	$273.9	$302.1	$344.1	$359.6
guanylyl cyclase c	$273.8	$342.5	$464.3	$601.5
HCV NS5B	$263.9	$336.3	$476.4	$658.7
Microsomal triglyceride transfer protein	$235.8	$281.4	$360.4	$451.8
proprotein convertase subtilisin kexin type 9	$226.4	$256.5	$305.9	$357.8
sodium glucose transporter	$220.6	$262.3	$332.5	$408.2
Polymerase Acidic Endonuclease	$198.2	$253.7	$360.5	$500.0
Dabigatran	$183.4	$202.1	$229.7	$254.3
aminolevulinate synthase	$178.0	$237.7	$355.8	$514.0
tissue nonspecific alkaline phosphatase	$161.7	$193.2	$248.5	$304.0
Tripeptidyl-peptidase	$156.6	$192.4	$255.9	$332.0
carboxypeptidase G	$151.8	$180.8	$230.1	$286.4
carbamoyl-phosphate synthase	$100.0	$117.9	$147.2	$179.0
Escherichia coli ATP synthase	$97.8	$117.2	$150.2	$187.1
(glucagon-like peptide 2 or glp2 or glp-2)	$86.4	$98.6	$118.5	$138.9
slamf7	$85.0	$96.0	$113.4	$131.7
phenylalanine ammonia	$71.2	$99.4	$157.5	$238.4
N Acetylgalactosamine 6 sulfatase	$10.7	$12.4	$15.1	$18.2

Values in millions with inflation adjustment to 2018. Discount rates or cost of capital calculated from year of NIH funding to year of approval of first to target product.

© 2023 Galkina Cleary E et al. *JAMA Health Forum.*

eTable 3. NIH costs for basic and applied research related to NMEs approved 2010-2019 without outlier elimination

	**Discount Rate[a]**			**Cost of Capital[a]**
	0%	3%	7%	10.5%
**Average NIH cost to launch of first drug with novel target (n=86)[b]**				
Basic and applied research, phased development failures, mean (SD)[c]	$2,330.7 ($5,113.1)	$2,860.6 ($6,490.5)	$3,831.1 ($9,126.0)	$5,030.2 ($12,205.6)
**NIH costs per drug (no spillovers)**				
Basic research on drug target, mean (SD)[d]	$2,235.6 ($5,093.1)	$2,751.2 ($6,467.2)	$3,697.5 ($9,097.0)	$4,868.9 ($12,147.2)
Applied research on approved drug, mean (SD)[e]	$154.8 ($536.8)	$187.3 ($673.1)	$246.3 ($928.7)	$318.7 ($1253.3)
Phased development failures candidate compounds[f]	$75.4	$80.6	$88.6	$96.8
Estimated total NIH cost (no spillovers)	$2,465.8	$3,019.1	$4,032.4	$5,284.4
**NIH costs per drug (with spillovers)**				
Basic research on novel drug target[g]	$784.4	$965.3	$1,297.4	$1,708.4
Estimated total NIH cost	$1,014.6	$1,233.3	$1,632.3	$2,123.9

[a]Discount rates calculated on years before drug approval. 3% and 7% Discount Rates are typically used to assess government investment. The 10.5% cost of capital is typically used to estimate industry costs of drug development. [b]The first-to-target drug is the first FDA approved product associated with a novel biological target. [c]Average NIH cost of published basic or applied research for 87 first-to target drugs. [d]Average NIH cost of published basic research on novel drug targets (n=86) to year of first drug approval. [e]Average cost for published applied research on drugs (n=356) approved 2010-2019. [f]Average NIH funding for phased trials of failed drugs. [g]Average cost per drug assuming 2.85 drugs associated with each biological target.

© 2023 Galkina Cleary E et al. *JAMA Health Forum.*

eTable 4. NIH costs associated with 356 drugs approved 2010-2019 with quartile and 95% percentile values

Percentile	0%	3%	7%	10.5%
25%	$358.8	$445.0	$556.9	$668.3
50%	$827.6	$1,005.4	$1,339.6	$1,664.6
75%	$72.1	$77.5	$92.8	$108.7
95%	$705.2	$839.5	$1,070.8	$1,337.0
**Brand name (generic name)**	**0%**	**3%**	**7%**	**10.5%**
Giapreza (angiotensin II)	$6,417.0	$8,189.4	$11,557.3	$15,890.0
Natpara (parathyroid hormone)	$3,621.1	$4,406.1	$5,810.4	$7,505.0
Esperoct (turoctocog alfa pegol)	$2,530.1	$3,318.3	$4,853.8	$6,884.7
Imlygic (talimogene laherparepvec)	$2,453.0	$3,074.2	$4,214.3	$5,622.3
Vonvendi (vonicog alfa)	$2,444.6	$3,023.3	$4,068.4	$5,338.3
Afstyla (lonoctocog alfa)	$2,175.4	$2,688.9	$3,622.3	$4,768.5
Kovaltry (Factor VIII)	$2,175.4	$2,688.9	$3,622.3	$4,768.5
Adynovate (rurioctocog alfa pegol)	$2,059.8	$2,495.7	$3,271.4	$4,202.6
Nuwiq (simoctocog alfa)	$2,055.7	$2,490.7	$3,265.2	$4,195.0
Obizur (susoctocog alfa)	$1,838.1	$2,200.2	$2,833.0	$3,577.3
Novoeight (turoctocog alfa)	$1,602.1	$1,899.5	$2,410.3	$2,999.3
Rebinyn (nonacog beta pegol)	$1,546.5	$2,022.8	$2,962.8	$4,221.0
Idelvion (albutrepenonacog alfa)	$1,466.3	$1,883.1	$2,687.4	$3,737.5
Ixinity (trenonacog alfa)	$1,353.3	$1,714.7	$2,397.5	$3,267.8
Kybella (deoxycholic acid)	$1,113.0	$1,384.7	$1,899.7	$2,559.1
Rixubis (nonacog gamma)	$1,105.2	$1,370.5	$1,850.8	$2,434.9
Andexxa (andexanet alfa)	$887.9	$1,157.5	$1,691.3	$2,408.3
Neutroval (filgrastim)	$742.1	$908.9	$1,203.0	$1,549.6
Glassia (alpha-1 antitrypsin)	$692.9	$816.3	$1,026.7	$1,266.2
Padcev (enfortumab vedotin)	$587.0	$668.2	$795.7	$928.4
Polivy (polatuzumab vedotin)	$587.0	$668.2	$795.7	$928.4
Nulojix (belatacept)	$575.6	$701.0	$928.4	$1,203.7
Farydak (panobinostat)	$527.2	$580.0	$659.5	$739.1
Genvoya (cobicistat+elvitegravir+tenofovir alafenamide+emtricitabine)	$486.5	$546.3	$638.9	$733.5

© 2023 Galkina Cleary E et al. *JAMA Health Forum.*

Cholbam (cholic acid)	$442.7	$562.8	$788.3	$1,073.0
Lynparza (olaparib)	$353.1	$381.5	$422.7	$462.2
Zinbryta (daclizumab)	$347.7	$423.5	$557.0	$715.7
Epidiolex (cannabidiol)	$290.6	$345.2	$442.4	$560.3
Ampyra (dalfampridine)	$276.0	$329.7	$419.6	$519.9
Venclexta (venetoclax)	$264.8	$284.0	$311.2	$336.7
Tretten (catridecacog)	$258.3	$305.4	$385.5	$477.2
Piqray (alpelisib)	$252.0	$278.7	$318.5	$357.7
Rydapt (midostaurin)	$235.3	$283.5	$370.2	$475.7
Orkambi (lumacaftor + ivacaftor)	$234.3	$261.7	$302.8	$343.6
Ibrance (palbociclib)	$232.4	$254.1	$286.7	$319.3
Nerlynx (neratinib)	$221.8	$248.5	$291.9	$339.1
Opdivo (nivolumab)	$214.8	$230.3	$252.6	$273.6
Yervoy (ipilimumab)	$200.9	$214.8	$234.6	$253.2
Pretomanid (pretomanid)	$199.5	$240.4	$307.4	$380.3
Kalydeco (ivacaftor)	$195.7	$210.8	$232.0	$251.7
Xpovio (selinexor)	$187.0	$210.9	$247.1	$283.4
Unituxin (dinutuximab)	$186.5	$221.7	$281.9	$351.8
Yondelis (trabectedin)	$182.2	$218.8	$284.4	$364.0
Zaltrap (aflibercept)	$172.9	$182.7	$196.5	$209.5
Turalio (pexidartinib)	$168.7	$189.4	$220.4	$251.1
Empliciti (elotuzumab)	$163.7	$175.3	$192.0	$207.9
Ruconest (conestat alfa)	$156.8	$191.4	$251.2	$320.5
Talzenna (talazoparib)	$153.1	$165.9	$184.5	$202.4
Mektovi (binimetinib)	$148.7	$167.2	$195.1	$222.7
Voraxaze (glucarpidase)	$144.1	$170.1	$214.2	$263.9
Gilenya (fingolimod hydrochloride)	$143.1	$161.4	$190.3	$220.7
Zydelig (idelalisib)	$142.6	$152.7	$167.3	$181.1
Ocaliva (obeticholic acid)	$126.6	$135.2	$147.7	$159.6
Sylvant (siltuximab)	$124.1	$137.2	$156.5	$175.4
Perjeta (pertuzumab)	$119.8	$131.4	$149.2	$167.4
Tivicay (dolutegravir)	$119.1	$130.9	$147.9	$164.2
Tecentriq (atezolizumab)	$118.5	$123.8	$131.0	$137.6

© 2023 Galkina Cleary E et al. *JAMA Health Forum.*

Gilotrif (afatinib)	$118.3	$129.3	$145.5	$161.4
Vizimpro (dacomitinib)	$117.4	$131.7	$154.3	$178.2
Tafinlar (dabrafenib)	$116.8	$123.2	$132.1	$140.3
Rubraca (rucaparib)	$114.9	$127.0	$145.4	$163.9
Tavalisse (fostamatinib disodium)	$113.6	$134.4	$167.3	$202.0
Imbruvica (ibrutinib)	$113.2	$119.6	$128.4	$136.5
Kisqali (ribociclib)	$112.8	$119.3	$128.4	$136.9
Inrebic (fedratinib)	$111.8	$139.4	$187.8	$244.3
Vitrakvi (larotrectinib)	$110.5	$116.4	$124.8	$132.6
Bosulif (bosutinib)	$110.4	$120.5	$135.6	$150.2
Caprelsa (vandetanib)	$110.0	$119.5	$133.5	$147.3
Lumoxiti (moxetumomab pasudotox)	$109.1	$126.2	$153.7	$183.1
Erleada (apalutamide)	$107.8	$121.4	$142.3	$163.6
Erwinaze (crisantaspase)	$105.5	$148.0	$230.4	$336.1
Pomalyst (pomalidomide)	$102.1	$110.1	$121.8	$133.1
Sirturo (bedaquiline)	$101.8	$109.1	$119.4	$129.0
Copiktra (duvelisib)	$98.4	$106.9	$119.5	$132.0
Beleodaq (belinostat)	$98.1	$106.7	$119.5	$132.0
Rozlytrek (entrectinib)	$97.4	$106.3	$119.3	$131.7
Cinqair (reslizumab)	$96.6	$111.2	$133.8	$156.8
Kyprolis (carfilzomib)	$96.1	$102.1	$110.5	$118.5
Mekinist (trametinib)	$92.6	$97.1	$103.4	$109.1
Nucala (mepolizumab)	$91.4	$106.0	$129.1	$153.6
Zelboraf (vemurafenib)	$89.2	$93.5	$99.4	$104.9
Blincyto (blinatumomab)	$87.8	$93.0	$100.1	$106.6
Xalkori (crizotinib)	$85.1	$89.2	$94.9	$100.1
Erivedge (vismodegib)	$80.2	$85.2	$92.1	$98.7
Emflaza (deflazacort)	$78.7	$93.9	$118.5	$145.1
Zytiga (abiraterone acetate)	$78.6	$82.5	$87.9	$92.8
Besponsa (inotuzumab ozogamicin)	$77.8	$86.4	$99.1	$111.7
Eylea (aflibercept ophthalmic solution)	$74.6	$79.0	$85.3	$91.2
Keytruda (pembrolizumab)	$73.9	$77.0	$81.2	$85.0
Asparlas (calaspargase pegol)	$71.5	$83.9	$103.2	$123.0

© 2023 Galkina Cleary E et al. *JAMA Health Forum.*

Bavencio (avelumab)	$67.2	$70.5	$75.0	$79.1
Daklinza (daclatasvir)	$64.7	$70.1	$78.1	$85.7
Xeljanz (tofacitinib)	$61.9	$66.8	$73.8	$80.4
Inlyta (axitinib)	$61.7	$66.5	$73.5	$80.5
Ninlaro (ixazomib citrate)	$61.6	$67.1	$75.2	$82.9
Nourianz (istradefylline)	$61.4	$76.4	$103.7	$137.0
Verzenio (abemaciclib)	$60.4	$63.2	$67.0	$70.5
Ofev (nintedanib)	$60.2	$63.0	$66.8	$70.3
Odomzo (sonidegib)	$60.0	$65.9	$74.7	$83.2
Defitelio (defibrotide)	$58.1	$62.8	$70.0	$77.5
Xtandi (enzalutamide)	$57.4	$60.7	$65.3	$69.6
Trogarzo (ibalizumab)	$54.4	$70.2	$97.8	$129.9
Cotellic (cobimetinib)	$53.8	$56.9	$61.3	$65.2
Edurant (rilpivirine)	$53.6	$56.7	$61.1	$65.1
Firazyr (icatibant)	$53.4	$67.2	$91.5	$120.2
Olumiant (baricitinib)	$52.2	$55.4	$60.1	$64.7
Northera (droxidopa)	$52.1	$63.4	$83.8	$108.6
Calquence (acalabrutinib)	$51.9	$54.1	$57.0	$59.6
Xuriden (uridine triacetate)	$51.8	$66.3	$92.1	$122.5
TPOXX (tecovirimat)	$51.3	$69.8	$105.4	$150.8
Impavido (miltefosin)	$49.7	$57.3	$70.0	$83.9
Corlanor (ivabradine)	$49.1	$53.8	$60.9	$68.1
Xiaflex (collagenase Clostridium histolyticum)	$48.6	$53.2	$60.2	$67.4
Ferriprox (deferiprone)	$48.3	$57.8	$73.9	$92.3
Braftovi (encorafenib)	$46.9	$50.5	$55.6	$60.3
Lucemyra (lofexidine)	$46.6	$60.7	$86.7	$119.0
Myalept (metreleptin)	$45.7	$51.1	$59.1	$66.8
Symdeko (ivacaftor + tezacaftor)	$45.4	$48.9	$54.1	$59.0
Rapivab (peramivir)	$44.9	$55.0	$72.8	$93.8
Lenvima (lenvatinib)	$44.2	$46.2	$49.0	$51.5
Esbriet (pirfenidone)	$43.5	$50.2	$60.8	$72.1
Gazyva (obinutuzumab)	$43.4	$45.9	$49.3	$52.5
Iclusig (ponatinib)	$43.0	$45.0	$47.9	$50.4

© 2023 Galkina Cleary E et al. *JAMA Health Forum.*

Picato (ingenol mebutate)	$42.6	$46.5	$51.9	$57.0
Tecfidera (dimethyl fumarate)	$42.3	$48.8	$59.4	$70.8
Benlysta (belimumab)	$42.2	$45.2	$49.5	$53.6
Fasenra (benralizumab)	$41.5	$48.6	$59.6	$70.9
Darzalex (daratumumab)	$41.0	$43.9	$48.0	$51.7
Eloctate (efraloctocog alfa)	$41.0	$44.0	$48.3	$52.3
Kadcyla (trastuzumab emtansine)	$40.7	$42.7	$45.3	$47.7
Jakafi (ruxolitinib)	$40.0	$42.7	$46.3	$49.7
Zejula (niraparib)	$39.8	$44.0	$50.4	$56.5
Cyramza (ramucirumab)	$39.3	$41.8	$45.4	$48.8
Prevymis (letermovir)	$37.2	$39.8	$43.6	$47.2
Galafold (migalastat hydrochloride)	$37.1	$48.6	$69.2	$93.6
Kengreal (cangrelor tetrasodium)	$36.2	$44.2	$58.3	$74.9
Imfinzi (durvalumab)	$36.0	$37.7	$40.1	$42.3
Vimizim (elosulfase alfa)	$34.8	$36.6	$39.0	$41.2
Elzonris (tagraxofusp)	$33.9	$36.9	$41.3	$45.5
Alprolix (eftrenonacog alfa)	$33.4	$37.3	$43.0	$48.5
Lumizyme (alglucosidase alfa)	$33.1	$35.4	$38.6	$41.7
Lorbrena (lorlatinib)	$32.8	$34.7	$37.3	$39.8
Ella (ulipristal acetate)	$32.1	$37.5	$46.9	$58.1
Firdapse (amifampridine phosphate)	$31.9	$36.3	$43.8	$52.1
Daurismo (glasdegib)	$31.6	$34.2	$37.9	$41.3
Eliquis (apixaban)	$31.3	$33.0	$35.4	$37.5
Tagrisso (osimertinib)	$30.7	$32.6	$35.2	$37.7
Osphena (ospemifene)	$30.1	$35.9	$45.4	$55.8
Trikafta (ivacaftor + tezacaftor+ elexacaftor)	$30.0	$31.5	$33.7	$35.7
Evenity (romosozumab)	$29.6	$31.8	$35.1	$38.4
Strensiq (asfotase alfa)	$28.9	$30.4	$32.5	$34.4
Moxidectin (moxidectin)	$28.9	$34.6	$45.5	$59.6
Incivek (telaprevir)	$27.4	$28.9	$31.1	$33.2
Radicava (edaravone)	$27.3	$33.2	$43.3	$54.8
Duavee (bazedoxifene acetate+Premarin)	$27.1	$30.1	$34.8	$39.8
Harvoni (sofosbuvir + ledipasvir)	$26.3	$29.3	$33.6	$37.8

© 2023 Galkina Cleary E et al. *JAMA Health Forum.*

Prolia (denosumab)	$25.6	$27.0	$29.0	$30.8
Vyndaqel (tafamidis)	$24.8	$28.0	$32.9	$37.9
Xermelo (telotristat)	$23.2	$24.7	$26.8	$28.8
Cometriq (cabozantinib)	$22.9	$24.3	$26.2	$28.0
Trulicity (dulaglutide)	$22.9	$24.8	$27.7	$30.3
Diacomit (stiripentol)	$22.1	$27.8	$37.3	$47.9
Brukinsa (zanubrutinib)	$22.0	$23.0	$24.4	$25.6
Nesina (alogliptin benzoate)	$21.7	$23.3	$25.7	$28.0
Provenge (sipuleucel-T)	$20.8	$22.3	$24.6	$26.9
Taltz (ixekizumab)	$20.7	$22.5	$25.1	$27.7
Dupixent (dupilumab)	$20.6	$24.9	$31.8	$39.1
Kanuma (sebelipase alfa)	$20.1	$21.8	$24.3	$26.5
Xospata (gilteritinib)	$19.4	$20.6	$22.2	$23.6
Ocrevus (ocrelizumab)	$19.4	$22.7	$27.9	$33.2
Mepsevii (vestronidase alfa)	$19.4	$21.2	$23.8	$26.2
Doptelet (avatrombopag)	$19.1	$23.6	$31.0	$39.1
Aliqopa (copanlisib)	$19.1	$21.4	$24.8	$28.0
Sovaldi (sofosbuvir)	$18.2	$19.5	$21.3	$23.0
Lonsurf (trifluridine + tipiracil hydrochloride)	$18.0	$18.5	$19.2	$19.9
Addyi (flibanserin)	$17.9	$18.6	$19.5	$20.4
Tibsovo (ivosidenib)	$17.8	$18.3	$19.0	$19.7
Nuplazid (pimavanserin tartrate)	$17.0	$18.6	$21.1	$23.7
Reblozyl (luspatercept)	$16.7	$17.7	$19.1	$20.3
Lartruvo (olaratumab)	$16.7	$17.3	$18.2	$19.0
Signifor (pasireotide)	$16.7	$18.2	$20.4	$22.5
Zinplava (bezlotoxumab)	$16.6	$17.4	$18.4	$19.4
Asclera (polidocanol)	$16.5	$20.0	$25.6	$31.7
Xadago (safinamide mesylate)	$16.4	$20.3	$26.8	$34.2
Skyrizi (risankizumab)	$15.9	$17.4	$19.5	$21.5
Stivarga (regorafenib)	$15.8	$16.6	$17.7	$18.7
Alecensa (alectinib hydrochloride)	$15.8	$16.9	$18.6	$20.1
Balversa (erdafitinib)	$15.8	$16.6	$17.9	$19.2
Motegrity (prucalopride succinate)	$15.6	$20.0	$29.2	$42.4

© 2023 Galkina Cleary E et al. *JAMA Health Forum.*

Otezla (apremilast)	$15.1	$16.1	$17.5	$18.8
Belviq (lorcaserin hydrochloride)	$14.1	$15.2	$16.8	$18.2
Xarelto (rivaroxaban)	$14.0	$14.8	$15.8	$16.7
Victrelis (boceprevir)	$13.7	$14.8	$16.4	$18.0
Siliq (brodalumab)	$13.2	$13.9	$14.7	$15.5
Opsumit (macitentan)	$12.7	$13.9	$15.5	$17.1
Entyvio (vedolizumab)	$12.6	$13.1	$13.7	$14.2
Juxtapid (lomitapide)	$12.6	$13.7	$15.3	$16.8
Zykadia (ceritinib)	$12.5	$13.1	$13.9	$14.6
Exondys 51 (eteplirsen)	$12.2	$14.5	$18.0	$21.7
Farxiga (dapagliflozin)	$11.9	$12.7	$13.9	$15.0
Poteligeo (mogamulizumab-kpkc)	$11.8	$13.3	$15.4	$17.5
Ilumya (tildrakizumab)	$11.8	$12.3	$13.1	$13.7
Zontivity (vorapaxar)	$11.5	$12.4	$13.8	$15.1
Yescarta (axicabtagene ciloleucel)	$10.9	$11.2	$11.6	$12.0
Austedo (deuterated tetrabenazine)	$10.1	$10.5	$10.9	$11.3
Victoza (liraglutide)	$10.1	$12.0	$15.1	$18.6
Kevzara (sarilumab)	$9.8	$10.6	$11.7	$12.7
Adcetris (brentuximab vedotin)	$9.2	$9.8	$10.6	$11.3
Vascepa (ethyl icosapentate)	$9.1	$10.4	$12.5	$14.6
Linzess (linaclotide acetate)	$8.9	$9.9	$11.5	$13.1
Sunosi (solriamfetol)	$8.8	$9.2	$9.8	$10.4
Adlyxin (lixisenatide)	$8.4	$9.4	$10.9	$12.5
Scenesse (afamelanotide)	$8.3	$9.4	$11.0	$12.5
Brilinta (ticagrelor)	$8.1	$8.6	$9.1	$9.7
Savaysa (edoxaban)	$8.1	$8.4	$8.9	$9.4
Zulresso (brexanolone)	$8.0	$8.4	$8.9	$9.4
Viekira Pak (ombitasvir + paritaprevir + ritonavir + dasabuvir)	$8.0	$8.2	$8.5	$8.8
Annovera (Nestorone + ethinylestradiol vaginal ring)	$7.8	$8.7	$10.1	$11.5
Cosentyx (secukinumab)	$7.8	$8.3	$9.0	$9.7
Arcapta neohaler (indacaterol maleate)	$7.4	$7.8	$8.5	$9.1
Actemra (tocilizumab)	$7.3	$7.8	$8.5	$9.2
Cerdelga (eliglustat)	$7.0	$7.7	$8.7	$9.7

© 2023 Galkina Cleary E et al. *JAMA Health Forum.*

Entresto (sacubitril + valsartan)	$6.1	$6.6	$7.2	$7.8
Tremfya (guselkumab)	$6.0	$6.2	$6.5	$6.7
Pradaxa (dabigatran etexilate)	$5.8	$6.0	$6.2	$6.4
Potiga (ezogabine)	$5.7	$6.4	$7.6	$8.8
Surfaxin (sinapultide)	$5.6	$6.0	$6.7	$7.3
Jardiance (empagliflozin)	$5.5	$5.6	$5.8	$6.0
Briviact (brivaracetam)	$5.2	$5.6	$6.1	$6.7
Alunbrig (brigatinib)	$5.0	$5.3	$5.8	$6.2
Krintafel (tafenoquine)	$4.9	$5.3	$5.8	$6.3
Zepatier (elbasvir + grazoprevir)	$4.4	$4.9	$5.7	$6.5
Natroba (spinosad)	$4.3	$4.6	$5.1	$5.6
Vraylar (cariprazine)	$4.3	$4.9	$6.0	$7.0
Invokana (canagliflozin)	$4.1	$4.4	$4.7	$5.1
Daliresp (roflumilast)	$4.0	$5.0	$6.7	$8.7
Aubagio (teriflunomide)	$4.0	$4.2	$4.4	$4.7
Pifeltro (doravirine)	$3.9	$4.2	$4.5	$4.9
Zioptan (tafluprost)	$3.9	$4.3	$4.9	$5.5
Fulyzaq (crofelemer)	$3.8	$4.3	$4.9	$5.5
Tymlos (abaloparatide)	$3.7	$3.9	$4.2	$4.4
Trulance (plecanatide)	$3.6	$3.7	$3.9	$4.1
Ozempic (semaglutide)	$3.5	$3.6	$3.8	$3.9
Zurampic (lesinurad)	$3.4	$3.5	$3.6	$3.7
Oxbryta (voxelotor)	$3.0	$3.1	$3.2	$3.4
Jetrea (ocriplasmin)	$2.9	$3.8	$5.2	$7.0
Mayzent (siponimod)	$2.9	$3.3	$4.0	$4.7
Spinraza (nusinersen)	$2.9	$3.1	$3.4	$3.6
Rhopressa (netarsudil)	$2.7	$2.9	$3.1	$3.3
Bridion (sugammadex sodium)	$2.6	$3.3	$4.4	$5.6
Repatha (evolocumab)	$2.6	$2.8	$3.0	$3.3
Xiidra (lifitegrast)	$2.6	$2.9	$3.3	$3.7
Olysio (simeprevir)	$2.5	$2.9	$3.4	$4.0
Biktarvy (bictegravir+emtricitibine+tenofovir alafenamide)	$2.4	$2.5	$2.6	$2.7
Zolgensma (onasemnogene abeparvovec)	$2.3	$2.4	$2.5	$2.6

© 2023 Galkina Cleary E et al. *JAMA Health Forum.*

Krystexxa (pegloticase)	$2.2	$2.7	$3.7	$4.7
Halaven (eribulin mesylate)	$2.2	$2.4	$2.7	$3.1
Wakix (pitolisant)	$2.1	$2.2	$2.4	$2.5
Egrifta (tesamorelin acetate)	$2.0	$2.2	$2.5	$2.8
Ajovy (fremanezumab)	$1.9	$2.1	$2.3	$2.5
Praluent (alirocumab)	$1.9	$2.0	$2.1	$2.2
Onpattro (patisiran)	$1.9	$1.9	$2.0	$2.1
Dalvance (dalbavancin)	$1.9	$2.2	$2.7	$3.3
Tanzeum (albiglutide)	$1.6	$1.7	$1.9	$2.0
Omontys (peginesatide)	$1.6	$1.6	$1.7	$1.8
Adakveo (crizanlizumab)	$1.4	$1.6	$2.0	$2.4
Kymriah (tisagenlecleucel-t)	$1.4	$1.4	$1.5	$1.6
Tresiba (insulin degludec)	$1.4	$1.5	$1.8	$2.0
Ibsrela (tenapanor hydrochloride)	$1.2	$1.3	$1.5	$1.6
Movantik (naloxegol)	$1.2	$1.2	$1.2	$1.3
Xofluza (baloxavir marboxil)	$1.2	$1.2	$1.3	$1.3
Xeomin (incobotulinumtoxinA)	$1.1	$1.2	$1.3	$1.5
Luxturna (voretigene neparvovec)	$1.1	$1.2	$1.3	$1.4
Belsomra (suvorexant)	$1.1	$1.2	$1.3	$1.4
Jevtana (cabazitaxel)	$1.0	$1.1	$1.1	$1.1
Cablivi (caplacizumab)	$0.8	$0.9	$1.0	$1.1
Rinvoq (upadacitinib)	$0.8	$0.9	$0.9	$0.9
Viibryd (vilazodone)	$0.8	$1.0	$1.5	$2.1
Bevyxxa (betrixaban)	$0.7	$0.9	$1.3	$1.6
Mavyret (glecaprevir + pibrentasvir)	$0.7	$0.8	$0.8	$0.8
Onfi (clobazam)	$0.7	$0.8	$1.0	$1.3
Vosevi (sofosbuvir + velpatasvir + voxilaprevir)	$0.7	$0.7	$0.7	$0.8
Vpriv (velaglucerase alfa)	$0.6	$0.6	$0.7	$0.7
Vyzulta (latanoprostene bunod)	$0.6	$0.6	$0.7	$0.7
Portrazza (necitumumab)	$0.5	$0.6	$0.7	$0.8
Praxbind (idarucizumab)	$0.5	$0.6	$0.6	$0.7
Steglatro (ertugliflozin)	$0.4	$0.4	$0.5	$0.5
Fycompa (perampanel)	$0.4	$0.4	$0.4	$0.5

© 2023 Galkina Cleary E et al. *JAMA Health Forum.*

Dayvigo (lemborexant)	$0.4	$0.4	$0.4	$0.4
Stendra (avanafil)	$0.4	$0.4	$0.4	$0.4
Crysvita (burosumab)	$0.4	$0.4	$0.4	$0.5
Symproic (naldemedine)	$0.3	$0.4	$0.4	$0.5
Viberzi (eluxadoline)	$0.3	$0.4	$0.4	$0.4
Edarbi (azilsartan medoxomil)	$0.3	$0.3	$0.3	$0.3
Tradjenta (BI-1356 BS (iv))	$0.3	$0.3	$0.3	$0.3
Adempas (riociguat)	$0.2	$0.3	$0.3	$0.3
Gattex (teduglutide)	$0.2	$0.2	$0.2	$0.2
Libtayo (cemiplimab)	$0.2	$0.2	$0.2	$0.2
Aptiom (eslicarbazepine acetate)	$0.1	$0.1	$0.2	$0.2
Epclusa (sofosbuvir + velpatasvir)	$0.1	$0.1	$0.1	$0.1
Fetzima (levomilnacipran)	$0.05	$0.05	$0.05	$0.05
Aimovig (erenumab)	-	-	-	-
Aklief (trifarotene)	-	-	-	-
Akynzeo (netupitant + palonosetron hydrochloride)	-	-	-	-
Akynzeo IV (fosnetupitant)	-	-	-	-
Anoro Ellipta (umeclidinium bromide + vilanterol)	-	-	-	-
Anthim (obiltoxaximab)	-	-	-	-
Aristada (aripiprazole lauroxil)	-	-	-	-
Beovu (brolucizumab)	-	-	-	-
Breo Ellipta (fluticasone furoate + vilanterol)	-	-	-	-
Brineura (cerliponase alfa)	-	-	-	-
Caplyta (lumateperone tosylate)	-	-	-	-
Carbaglu (carglumic acid)	-	-	-	-
Elelyso (taliglucerase alfa)	-	-	-	-
Emgality (galcanezumab)	-	-	-	-
Enhertu (trastuzumab deruxtecan)	-	-	-	-
Eucrisa (crisaborole)	-	-	-	-
Gamifant (emapalumab)	-	-	-	-
Givlaari (givosiran)	-	-	-	-
Hemlibra (emicizumab)	-	-	-	-
Hetlioz (tasimelteon)	-	-	-	-

© 2023 Galkina Cleary E et al. *JAMA Health Forum.*

Horizant (gabapentin enacarbil)	-	-	-	-
Idhifa (enasidenib)	-	-	-	-
Ingrezza (valbenazine)	-	-	-	-
Jeuveau (Prabotulinum toxin A)	-	-	-	-
Jivi (damoctocog alfa pegol)	-	-	-	-
Kynamro (mipomersen sodium)	-	-	-	-
Lastacaft (alcaftadine)	-	-	-	-
Latuda (lurasidone hydrochloride)	-	-	-	-
Mulpleta (lusutrombopag)	-	-	-	-
Myrbetriq (mirabegron)	-	-	-	-
Natazia (dienogest + estradiol valerate)	-	-	-	-
Nubeqa (darolutamide)	-	-	-	-
Orilissa (elagolix)	-	-	-	-
Oxervate (cenegermin)	-	-	-	-
Palynziq (pegvaliase)	-	-	-	-
Parsabiv (velcalcetide)	-	-	-	-
Plegridy (PEG-interferon ß1a)	-	-	-	-
Rexulti (brexpiprazole)	-	-	-	-
Reyvow (lasmiditan)	-	-	-	-
Striverdi Respimat (olodaterol)	-	-	-	-
Takhzyro (lanadelumab)	-	-	-	-
Tegsedi (inotersen)	-	-	-	-
Trintellix (vortioxetine)	-	-	-	-
Tudorza Pressair (aclidinium bromide)	-	-	-	-
Ubrelvy (ubrogepant)	-	-	-	-
Ultomiris (ravulizumab)	-	-	-	-
Uptravi (selexipag)	-	-	-	-
Varubi (rolapitant)	-	-	-	-
Vyleesi (bremelanotide acetate)	-	-	-	-
Vyondys 53 (golodirsen)	-	-	-	-
Xcopri (cenobamate)	-	-	-	-
Yupelri (revefenacin)	-	-	-	-
Veltassa (patiromer)	-	-	-	-

© 2023 Galkina Cleary E et al. *JAMA Health Forum.*

Prepopik (Picoprep)	-	-	-	-
Seysara (sarecycline)	-	-	-	-

Values in millions with inflation adjustment to 2018. Discount rates or cost of capital calculated from year of NIH funding to year of approval of first to target product.

© 2023 Galkina Cleary E et al. *JAMA Health Forum.*

eTable 5. Calculation of the NIH contribution to phased clinical trials of failed clinical compounds for each product approval

	Phase Transition rate[a]	# in phase/approval		Average NIH costs for phase (millions)[b]				Average NIH cost for failed candidates (millions)			
		#	# failed	0%	3%	7%	10.5%	0%	3%	7%	10.5%
**Phase 3 to approval**	56.0%	1.79	0.79	$3.9	$4.3	$4.8	$5.3	$3.1	$3.3	$3.8	$4.2
**Phase 2**	35.2%	5.08	4.08	$7.2	$7.6	$8.3	$8.9	$29.3	$31.0	$33.7	$36.3
**Phase 1**	59.5%	8.53	7.53	$5.7	$6.1	$6.8	$7.5	$43.0	$46.2	$51.2	$56.2
**Overall approval rate:**	11.7%	-	-	-	-	-	**Total costs of failed trials:**	$75.4	$80.6	$88.6	$96.8

Values in millions with inflation adjustment to 2018. Discount rates or cost of capital calculated from year of NIH funding to year of approval of first to target product. [a]Discount rates from DiMasi JA, et al. (DiMasi, Grabowski et al. 2016). [b]Phase specific NIH costs from: Zhou et al. (Zhou 2022).

© 2023 Galkina Cleary E et al. *JAMA Health Forum.*

eFigure 1. NIH funding for basic and applied research related to drugs approved 2010-2019 by Project Activity Code

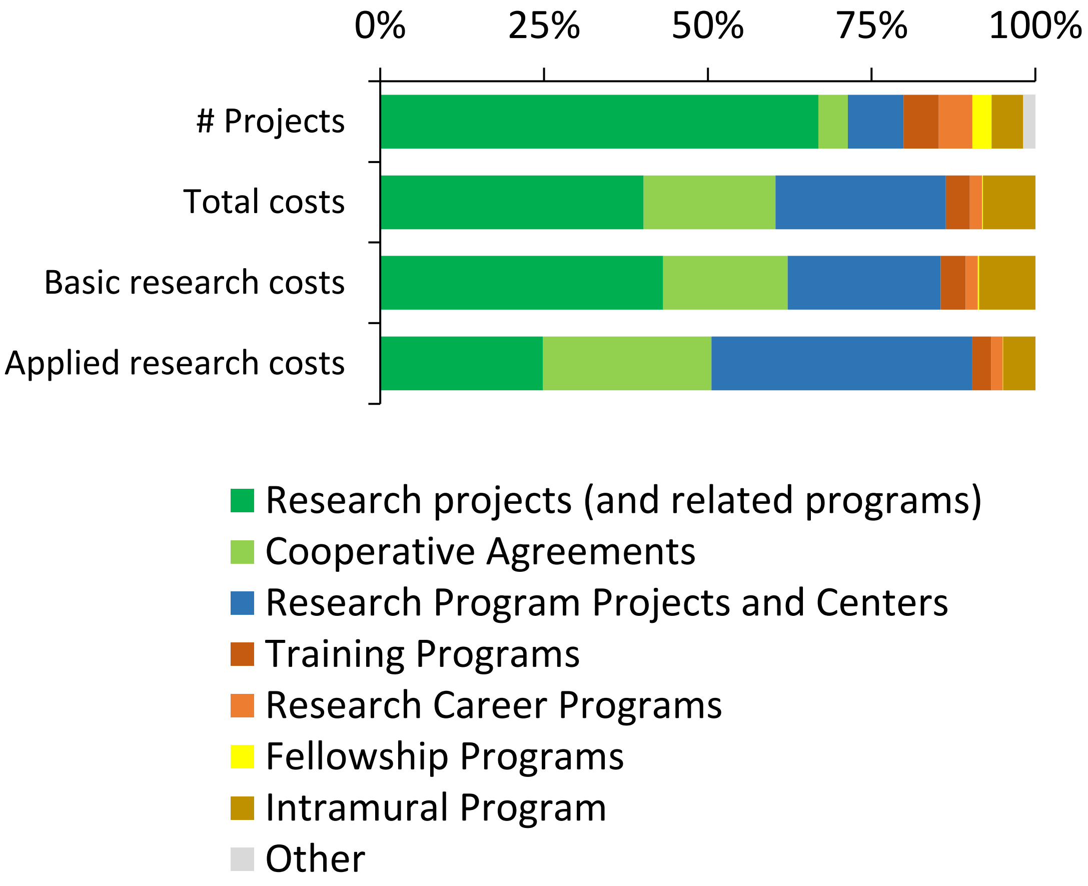

© 2023 Galkina Cleary E et al. *JAMA Health Forum.*

eFigure 2. Number of approved FDA drugs (through June 2015) associated with 515 drug targets

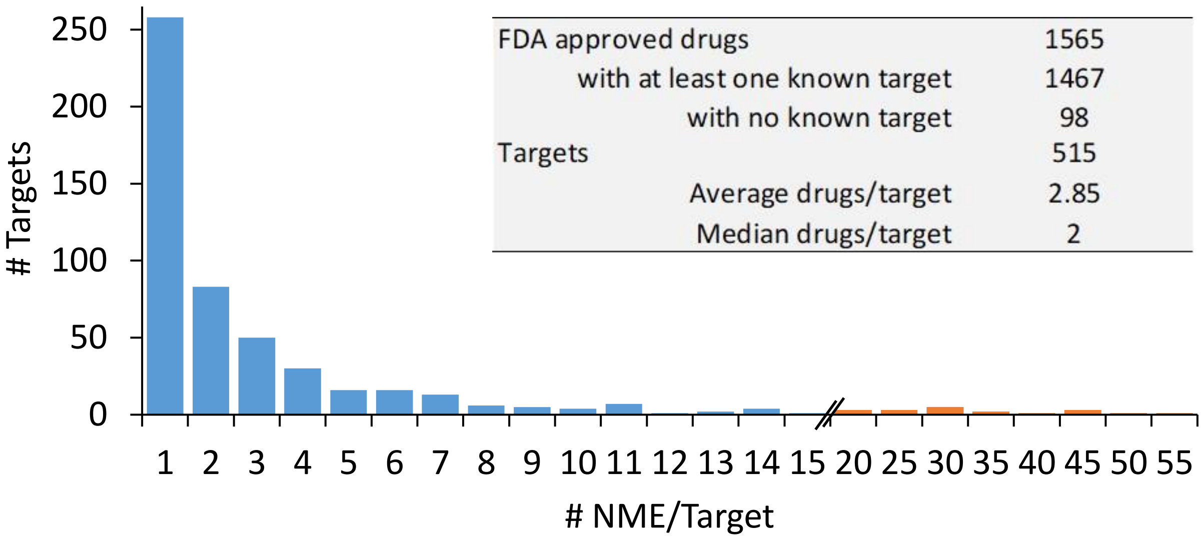

Data calculated after exclusion of products derived from blood or tissue, antimicrobials, and reformulations. Data on 1,467 drugs was recalculated from Santos R, et al. (Santos, Ursu et al. 2017).

© 2023 Galkina Cleary E et al. *JAMA Health Forum.*

eReferences

Boyack, K. W. and P. Jordan (2011). "Metrics associated with NIH funding: a high-level view." Journal of the American Medical Informatics Association 18(4): 423-431.

Cleary, E., M. J. Jackson and F. Ledley (2020). "Government as the First Investor in Biopharmaceutical Innovation: Evidence From New Drug Approvals 2010–2019." Institute for New Economic Thinking Working Paper Series(133).

Cleary, E. G., J. M. Beierlein, N. S. Khanuja, L. M. McNamee and F. D. Ledley (2018). "Contribution of NIH funding to new drug approvals 2010–2016." Proceedings of the National Academy of Sciences 115(10): 2329-2334.

Cleary, E. G. and F. D. Ledley (2020). "NIH funding for research underlying new cancer therapies." The Lancet Oncology 21(6): 755-757.

DiMasi, J. A., H. G. Grabowski and R. W. Hansen (2016). "Innovation in the pharmaceutical industry: new estimates of R&D costs." Journal of Health Economics 47: 20-33.

Eder, J., R. Sedrani and C. Wiesmann (2014). "The discovery of first-in-class drugs: origins and evolution." Nature Reviews Drug Discovery 13(8): 577-587.

Lanthier, M., K. L. Miller, C. Nardinelli and J. Woodcock (2013). "An improved approach to measuring drug innovation finds steady rates of first-in-class pharmaceuticals, 1987–2011." Health Affairs 32(8): 1433-1439.

OMB (1992). Circular A-94 Guidelines and Discount Rates for Benefit-Cost Analysis of Federal Programs. O. o. M. a. Budget.

OMB (2017). Circular A-4, Regulatory Review. O. o. M. a. Budget.

Santos, R., O. Ursu, A. Gaulton, A. P. Bento, R. S. Donadi, C. G. Bologa, A. Karlsson, B. Al-Lazikani, A. Hersey and T. I. Oprea (2017). "A comprehensive map of molecular drug targets." Nature reviews Drug discovery 16(1): 19-34.

Wouters, O. J., M. McKee and J. Luyten (2020). "Estimated Research and Development Investment Needed to Bring a New Medicine to Market, 2009-2018." JAMA 323(9): 844-853.

Zhou, E., Jackson, M.J., Ledley, F.D. (2022). "NIH Contribution to phased clinical development of drugs approved from 2010-2019: Tech Note." Center for Integration of Science and Industry, Bentley University.

Zhu, F., B. Han, P. Kumar, X. Liu, X. Ma, X. Wei, L. Huang, Y. Guo, L. Han and C. Zheng (2010). "Update of TTD: therapeutic target database." Nucleic acids research 38(suppl_1): D787-D791.

© 2023 Galkina Cleary E et al. *JAMA Health Forum.*
