# Supplementary material for: Comparison of Research Spending on New Drug Approvals by the National Institutes of Health vs the Pharmaceutical Industry, 2010-2019
Source: JAMA Health Forum. 2023 Apr 28;4(4):e230511. doi: 10.1001/jamahealthforum.2023.0511 (PMC10148199; doi:10.1001/jamahealthforum.2023.0511)
Supplement: Supplement 2. — Data sharing statement [file jamahealthforum-e230511-s002.pdf]

Data Sharing Statement

Galkina Cleary. Comparison of Research Spending on New Drug Approvals by the National Institutes of Health vs the Pharmaceutical Industry, 2010-2019. *JAMA Health Forum*. Published April 28, 2023. doi:10.1001/jamahealthforum.2023.0511

Data
Data available: Yes
Data types: Data (not involving human participants)
How to access data: All of the data is provided in the Supplement or freely available online through links provided in eMethods. Python code for replicating the core data collection is publicly available on GitHub and a public dashboard is available for those interesting in applying these methods. There are no restrictions on use of these data or methods.
When available: With publication

Supporting Documents
Document types: Statistical/analytic code
How to access documents: https://github.com/BentleySciIndustry/NIH-Contribution-to-phased-clinical-development-of-drugs-approved-Supplemental-Data-Sharing.git
When available: beginning date: 02-01-2023

Additional Information
Who can access the data: No restrictions
Types of analyses: No restrictions
Mechanisms of data availability: No restrictions (public URL)
Any additional restrictions: No restrictions
